# Estimating parametric phenotypes that determine anthesis date in *Zea mays*: Challenges in combining ecophysiological models with genetics

**DOI:** 10.1371/journal.pone.0195841

**Published:** 2018-04-19

**Authors:** Abhishes Lamsal, Stephen M. Welch, Jeffrey W. White, Kelly R. Thorp, Nora M. Bello

**Affiliations:** 1 Department of Agronomy, Kansas State University, 2104 Throckmorton Plant Science Center, Manhattan, KS, United States of America; 2 USDA-ARS Arid-Land Agricultural Research Center, Maricopa, AZ, United States of America; 3 Department of Statistics, Kansas State University, Manhattan, KS, United States of America; Clemson University, UNITED STATES

## Abstract

Ecophysiological crop models encode intra-species behaviors using parameters that are presumed to summarize genotypic properties of individual lines or cultivars. These genotype-specific parameters (GSP’s) can be interpreted as quantitative traits that can be mapped or otherwise analyzed, as are more conventional traits. The goal of this study was to investigate the estimation of parameters controlling maize anthesis date with the CERES-Maize model, based on 5,266 maize lines from 11 plantings at locations across the eastern United States. High performance computing was used to develop a database of 356 million simulated anthesis dates in response to four CERES-Maize model parameters. Although the resulting estimates showed high predictive value (R2 = 0.94), three issues presented serious challenges for use of GSP’s as traits. First (expressivity), the model was unable to express the observed data for 168 to 3,339 lines (depending on the combination of site-years), many of which ended up sharing the same parameter value irrespective of genetics. Second, for 2,254 lines, the model reproduced the data, but multiple parameter sets were equally effective (equifinality). Third, parameter values were highly dependent (*p*<10^−6919^) on the sets of environments used to estimate them (instability), calling in to question the assumption that they represent fundamental genetic traits. The issues of expressivity, equifinality and instability must be addressed before the genetic mapping of GSP’s becomes a robust means to help solve the genotype-to-phenotype problem in crops.

## Introduction

Finding methods to predict crop phenotypes from genotypes, often termed the *G2P* problem, is one of the highest priorities of applied biological research [1] and is central to addressing global food security issues that will otherwise become acute by mid-century [2]. Accurate G2P prediction will enable crop breeders to implement efficient crossing schemes that will increase annual rates of genetic gain in the targeted population of (perhaps novel) environments. In addition, when growing improved germplasm, farmers will be better able to predict the outcomes of alternative in-season management tactics and make decisions that have higher probabilities of favorable results in terms of productivity, profits, environmental impacts, and other criteria.

Many types of genetic resources have been used to dissect G2P relations. Nested association mapping (NAM) populations are large sets of lines (e.g., 5000 or more) developed by crossing schemes that convey great power to identify genomic regions with a quantifiable likelihood of influencing traits measured in field experiments [3]. For one such population in *Zea mays*, previous work located numerous genomic intervals of potential relevance to quantitative traits and suggested possible candidate genes in other species that might also be of importance [4]. Nonetheless, analysis of the maize NAM population has focused mainly on traits measured directly in the field or estimated through simple conversions, such as days to flowering expressed as thermal time [4,5]. Arguably, for accurate G2P prediction of complex traits, a more process-focused characterization of traits is required. This is particularly true for yield because more fundamental processes such as seed development and growth have rates that change dynamically during the season in response to conditions both internal and external to the plant.

One approach for analyzing dynamic crop phenotypes utilizes ecophysiological crop models (ECM’s) and quantitative genetics in a two-step process [6–10]. The first step is to use an ECM to describe the dynamic, environmentally responsive mechanisms that determine crop growth and development on daily or hourly time scales. To simulate genetically controlled differences in the behavior of individual genetic lines, ECM’s use numeric constants called genotype specific parameters (GSP’s). Because GSP’s largely encode environmental sensitivities and stress-free, near-optimum performance levels, they mediate phenotypic gene by environment (GxE) interactions. However, they are themselves expected to be constant across environments. The GSP’s thus are intended to represent physiologically more fundamental traits that have higher heritabilties and result in stronger trait-genetic marker associations than expected for directly measured complex phenotypes. The second step is to use quantitative genetic methods such as genomic prediction [11] to relate the GSP’s to genotypic markers [6].

The lynchpin of this approach is the accurate estimation of the GSP’s. Direct measurement of GSP’s is typically too time- and resource-demanding to be feasible for large numbers of lines and environmental conditions, as determined by location and growing season (i.e., site-years). Instead, crop data with simpler measurement protocols, for example yield or anthesis dates, can be used for GSP estimation through a computational process known as model inversion. During inversion, multiple trial values of GSP’s are generated under control of a suitable algorithm [12]. The trial values are used to project the behaviors of a given line, and optimum values of the GSP’s are determined by maximizing agreement with the corresponding field measurements.

Two problems that can reduce the effectiveness of ECM inversion are equifinality and GSP stability across environments. Equifinality occurs when multiple sets of parameter values generate identical ECM outputs [13,14]. Equifinality is a non-problem in situations where the only concern is whether model outputs match field measurements. However, when GSP values are intermediaries obtained with the intent to genetically map them, equifinality is very problematic. The linear quantitative genetic statistical models used in mapping employ allelic states (i.e., marker genotypes) as independent variables. Their dependent variables are phenotypes that grade seamlessly from traits that are usually measured directly (e.g., plant height) through GSPs that can either be measured or inferred (e.g., phyllochron interval and critical short day length) to ones that, for large numbers of lines, can in practice only be inferred (e.g., maximum *potential* seed size). When equifinality is present, the range of possible GSP values is wider, thus reducing or eliminating detection of significant genotypic effects. Unfortunately, many commonly used model inversion methods [15] only output a single, point GSP estimate, allowing equifinality to escape detection. Even when error bounds are determined [16], it may not be evident whether the cause is equifinality as opposed to biological variability. The potential for adverse effects of equifinality on genetic mapping warrants an investigation of its prevalence during estimation studies.

The stability of GSP estimates across the environments is also of concern. When an estimate varies with the set of environments used in model inversion, there is no way to determine which value to use when weather conditions, soil properties or crop management differ from those used in model training. Indeed, given that GSP’s are, as a defining property, assumed to be free of G×E interactions, the detection of instability is *prima facie* evidence that a particular parameter is not immediately usable as a GSP. Therefore, instability should be assessed as part of any GSP estimation protocol.

The overall goal of this study was to investigate the estimation of GSP’s controlling anthesis date in a large maize mapping population (> 5200 lines), applying an ECM inversion technique to data from eleven site-years of maize field experiments [5]. Not only is anthesis date a phenotype of major biological significance, but it was also studied in this same panel using conventional statistical genetic methods [4,17].

Parameter estimation is especially challenging when large numbers of lines are involved. Therefore, we sought a method that was efficient for large estimation tasks while explicitly supporting an investigation of equifinality and stability. Specific objectives were to 1) estimate CERES-Maize GSP’s for each maize NAM line, 2) assess the estimated GSP’s for evidence of equifinality and instability, and 3) examine the estimated GSP’s for other potential issues or opportunities.

## Materials and methods

### Experimental data

Anthesis date data for a total of 5266 maize lines were obtained from the Panzea data repository (http://www.panzea.org). The lines were members of three genetic panels. In particular, 4785 lines were from the 25 RIL panels comprising the maize NAM set described above; 200 RIL lines, referred as the IBM (**I**ntermating **B**73 x **M**o17) panel [18]; and a diversity panel [19] contained an additional 281 lines. Various combinations of these lines were grown at six sites in the United States, providing a total of 11 site-years during 2006 and 2007 (Table 1). For the NAM lines and IBM population, trials were arranged as augmented incomplete block designs, having one replication per trial. For each trial, lines were grouped by family with augmented incomplete blocks within each family. Each incomplete block contained 20 RILs and two checks, B73 and the second parent of the family. A similar design was used for the diversity panel. Anthesis date was recorded as the date on which 50% of the plants in a plot had begun shedding pollen. Data on daily maximum and minimum daily temperatures for each site were provided by the maize NAM collaborators [5]. These stations followed standard instrument and siting guidelines, and data were found comparable to 2.5 arc minutes (ca. 4 km) gridded data [20] (Figs A and B in S1 File and Discussion). As verified by inspecting model source code, the calculated photoperiods include civil twilight, defined as when the sun is <6° below the rising or setting horizon.

**Table 1:** Geographical coordinates, sowing dates, total number of lines planted and number of lines for which anthesis dates were observed for all site-year combinations.

	NY6*	NY7	NC6	NC7	MO6	MO7	IL6	IL7	FL6	FL7	PR6
Latitude (deg)	42.73	42.73	35.67	35.67	38.89	38.89	40.08	40.08	25.51	25.51	18.00
Longitude (deg)	-76.66	-76.66	-78.49	-78.49	-92.23	-92.23	-88.2	-88.2	-80.49	-80.49	-66.51
Sowing Date (DOY)	128	135	122	120	137	138	128	137	265	280	314
Number of lines sown	5478	5478	5478	5478	5478	5478	5478	5478	5026	3753	5131
Number of lines with data	4743	5236	5236	5160	3261	2555	5036	5178	4943	3742	4401

* “NY6” denotes 2006 planting in New York; abbreviations and for other site-years are formed analogously; NY = New York, NC = North Carolina, MO = Missouri, IL = Illinois, FL = Florida, PR = Puerto Rico.

### CERES-Maize model

The Crop Estimation through Resource and Environment Synthesis (CERES)-Maize v 4.5 [21,22] is one of the oldest and most widely used maize ECM’s. Four GSP’s (P1, P2, P2O, and PHINT; Table 2) that control time to anthesis were considered [23,24]. The interval from emergence through the end of the juvenile phase (Stage 1) is calculated by accumulating daily thermal time until the value of P1 is reached. Stage 2 follows immediately and lasts until tassel initiation. Stage 3 lasts from tassel initiation through anthesis date. Stage 2 lasts four days when the day length (including civil twilight) is less than the critical photoperiod, P2O, and otherwise depends on photoperiod response. P2 specifies the number of extra days spent in Stage 2 for every hour by which the day length exceeds P2O. The length of Stage 3 is controlled by leaf development. The model assumes that (1) there are five embryonic leaves; (2) two new leaves initiate during each phyllochron interval as measured in thermal time; and (3) anthesis date occurs when all leaves present at the end of Stage 2 (i.e., total leaf number, TOLN) are fully expanded. This happens on the day when the thermal time accumulation from the end of Stage 2 reaches (TOLN +0.5) × PHINT—SUMDTT.

**Table 2:** Parameter definitions, ranges, and number of unique values from Sobol sequence generation.

Parameter	Definition	Unit	Min	Max	No. of unique values
P1	Thermal time from seedling emergence to end of juvenile phase	GDD (°C)	150	450	30001
P2O	Critical Short day length below which day length does not affect development rate	h	10	14	401
P2	Extent to which development (expressed as days) is delayed for each hour increase in photoperiod above the longest photoperiod at which development proceeds at a maximum rate (i.e., P2O)	rate	0	2	20001
PHINT	Phyllochron interval (Interval between successive leaf tip appearances)	GDD (°C)	25	70	45001

The soil water and nutrient components, tillage, pest, and disease options, none of which affect anthesis date in CERES-Maize, were switched off during the simulation runs to reduce the computing time required. Row spacing and planting depth were set to 0.5 m and 2.5 cm, respectively.

### Parameter estimation

#### Search strategy

**Fig 1:**
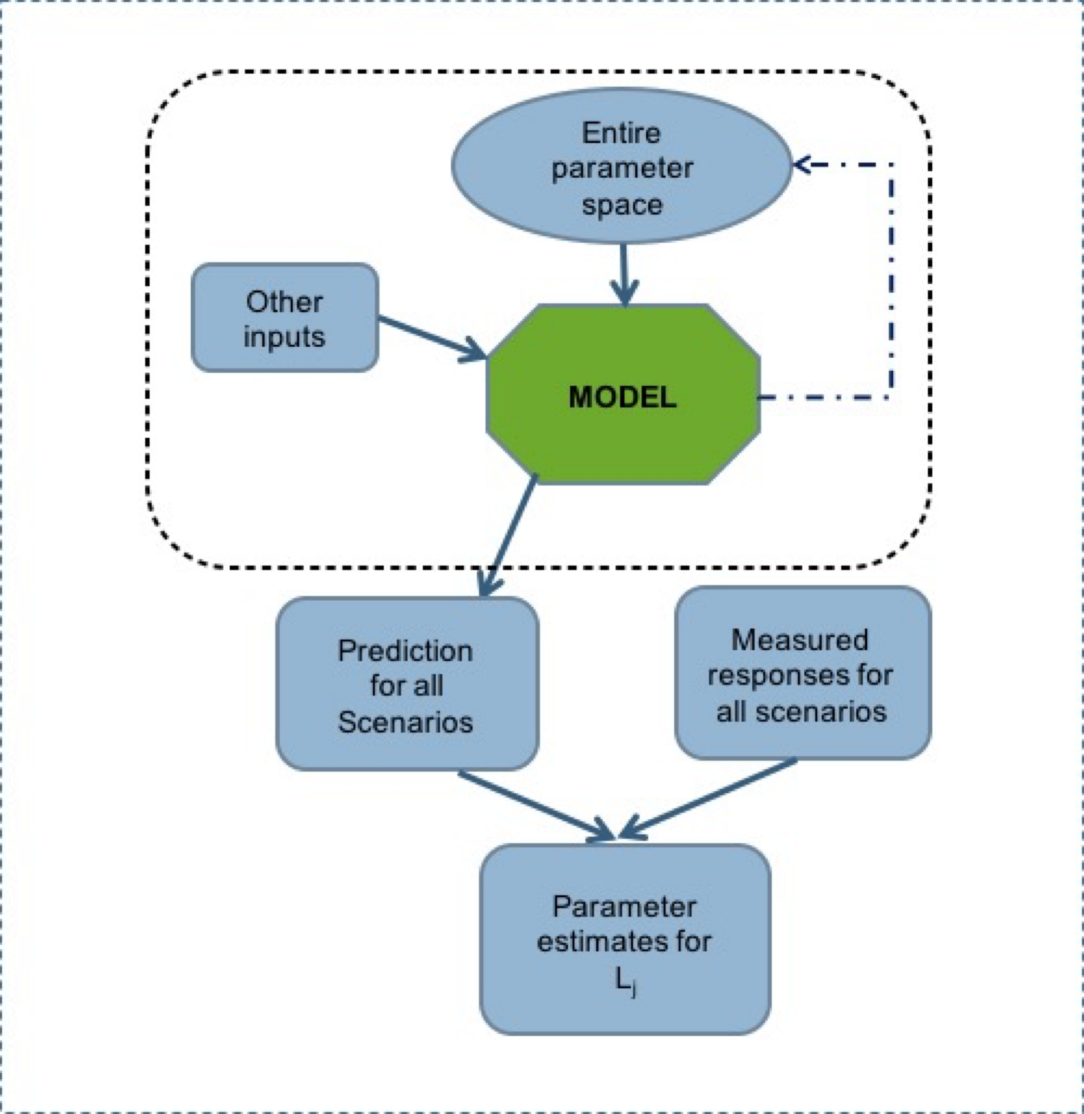
Parameter estimation using database method. L_1…N_ are the lines in the data set.

This study adapted a parameter space search algorithm developed by [25–27] (Fig 1). First, model simulations were run for each of the 11 site-years across a multidimensional set of parameter value combinations that were stored in a database along with the simulated anthesis dates. Second, for each line, the root mean square error (RMSE) [28] in days between observed and simulated anthesis dates as summed across site-years was calculated for every combination of parameter values. That is, for line *l*,

$${RMSE}_{l}=\sqrt{\frac{1}{n}\sum_{i=1}^{n}(Y_{p}-Y_{o}{)}^{2}}$$ (1)

where, *n* is the number of observations for that line (i.e., one per site-year), and *Y*_*p*_ (*Y*_*o*_) is the simulated (observed) anthesis date. For each line, the search engine then output any (ideally just one) parameter value combination that produced a minimal RMSE. The minimal RMSE criterion has also been used in GSP searches done by [29–32]. This paper calls this procedure the “Sobol database algorithm” after the method by which the parameter value combinations were produced. It is described in the next section.

#### Sampling the model parameter space with Sobol sequences

Unlike [25–27], who sampled the parameter space with a rectilinear grid, a Sobol sequence was used to avoid the combinatorial explosion in computational requirements that accompanies increasing dimensionality. Sobol sequences belong to a family of quasi-random processes that generate parameter sets samples dispersed as uniformly as possible over a multidimensional parameter space [33]. Sobol sequences offer reduced spatial variation compared to other sampling methods (e.g., random, stratified, Latin hypercube), making them more robust [34]. The Sobol algorithm was coded in Python and used to generate 32,400,070 GSP sets (Table 2). The resulting database had 356,400,700 entries, consisting of the CERES-Maize simulated anthesis dates for each of the 11 site-years x 32,400,770 GSP sets.

#### High performance computing

To execute the needed 356 million model runs, a simple wrapper was written that iteratively inserted the desired parameter values into the CERES-Maize input files. This is a very efficient procedure because, with minor modifications, the wrapper can be reused to analyze other CERES model parameters or outputs. The model runs were conducted using 112 processors on the “Stampede” supercomputer (https://www.tacc.utexas.edu/systems/stampede) at the Texas Advanced Computing Center (TACC), requiring 63,372 CPU hours. The predicted anthesis dates were transferred to the BeoCat computing cluster (https://support.beocat.ksu.edu/BeocatDocs/index.php/Compute_Nodes) at Kansas State University, where RMSE values were tabulated for each line × parameter value combination across all site-years. GSP combinations that gave the lowest RMSE values were recorded. This process took 7 h for all lines on 200 Xeon E5-2690 with ca. 15 minutes of wall clock time per line. An advantage of the database approach is that, if needs or more refined interests dictate, searches using completely different objective functions can be executed without the need for additional CPU-time-consuming model runs. Subsequently, when it was determined that specialized analyses were needed, a more labor-intensive, manual undertaking extracted the anthesis date submodel, ported it to Python and ran it independently on Beocat. Because such submodel extraction could easily introduce errors, checks were performed to insure that outputs of standalone code matched those of the full CERES-Maize model.

### Assessing estimate properties

#### Equifinality

The extent of equifinality for a line was quantified as one less than the number of Sobol parameter combinations that produced the identical, minimal RMSE value (i.e., the “number of ties”; see Table A in S2 File for an example tie). During the database tabulation, the “best combination of parameter estimates seen so far” was updated only if its RMSE value was strictly better than all previously evaluated ones. Thus, the first single estimate encountered giving a minimum RMSE was reported. This is referred to below as the “first-best-found” estimate. The number of subsequently examined estimates having the same RMSE as the first-best-found is the extent of equifinality.

#### Relationships among parameter estimates

In genetics, one expects to see trait correlations, the architecture of which is central to understanding and prediction. Thus, a possibility was that correlations among GSP estimates might reflect biologically important differences among populations. Scatter plots and Pearson correlations were used to examine the relations among parameters.

#### Testing for parameter stability across environments

To determine whether the GSP estimates depended on the particular set of environments used to obtain them, a novel statistical approach was developed. A subset of 539 lines was identified that were present in all 11 site-years. Next, all 330 mathematical combinations of the 11 site-years when chosen seven-at-a-time were constructed. The number seven was selected because preliminary Sobol database tabulations revealed that equifinality increased dramatically when fewer than seven site-years were used in estimation (see Results). We conducted 177,870 (= 539×330) line x environmental subset parameter searches. Because equifinality might reduce the power of the statistical test used to detect instability (next paragraph), 114,314 searches were discarded because they had ties. Of the 330 site-year subsets run, 297 were identified that had at least 100 lines remaining after ties were removed. Each of the 539 lines was present in at least 28 site-year subsets. By this process an overall total of 60,834 estimates were generated for each of the four GSP’s in the study.

The following statistical model was used to test for stability in parameter estimates across environmental subsets:

$$\rho_{l,e}=\mu_{\rho }+\alpha_{l}+\beta_{e}+\epsilon_{l,e}$$ (2)

where *ρ*_*l*,*e*_ represents an estimate of the GSP.*ρ* (i.e., either P1, P2, P2O, or PHINT) for the *l*^*th*^ line (*l* = 1,2,… 539) obtained from the *e*^*th*^ site-year subset (e = 1,2,… 297), μ is the intercept parameter, acting as an overall mean of GSP *ρ* across all lines and site-year subsets;*α*_*l*_ is the differential random effect of line *l*, assumed to be distributed $\alpha_{l}∼N(0,\sigma_{l}^{2})$; *β*_*e*_; is the differential random effect of the *e*^*th*^ subset of site-years, assumed to be distributed $\beta_{e}∼N(0,\sigma_{e}^{2})$ and *ε*_*l*,*e*_; and is the remaining residual unique to the *l*,*e*^*th*^ observed GSP estimate and assumed $\varepsilon_{l,e}∼NIID(0,\sigma_{\varepsilon }^{2})$ The differential line effects *α*_*l*_ are considered to be random, as is common in field studies of plant population biology. Further, the differential effects of site-year subsets, *β*_*e*_, were treated as random because the corresponding environmental subsets are combinations of 7 out of 11 plantings considered to be a representative, if not random, sample of the population of possible site-years to which we are interested in inferring.

If the estimation of any GSP parameter *ρ* were stable across the site-year subsets, one would expect the variance of *β*_*e*_, namely $\sigma_{e}^{2}$ to be zero; alternatively, if estimation is unstable, one would expect $\sigma_{e}^{2}>0$. To test this hypothesis set, two competing versions of the statistical model in Eq (2) were fit, one with and one without the random effect of site-year subsets *β*_*e*_ for each of the GSP’s *ρ* = P1,P2,P2O, and PHINT. For each GSP, the two competing models were compared using a likelihood ratio test statistic against a central chi-square distribution with half a degree of freedom to account for the fact that the test was conducted on the boundary of the parameter space. Statistical models were fit using the linear mixed-effects model package lmer in R [35] with optimization based on the log-likelihood option. The lmer package also calculated the Akaike and Bayesian Information Criteria (AIC [36] and BIC [37], respectively), which allowed additional assessments of fits for the statistical models that included or excluded the random effects of site-year subsets.

## Results

### Observations vs. simulations

**Fig 2:**
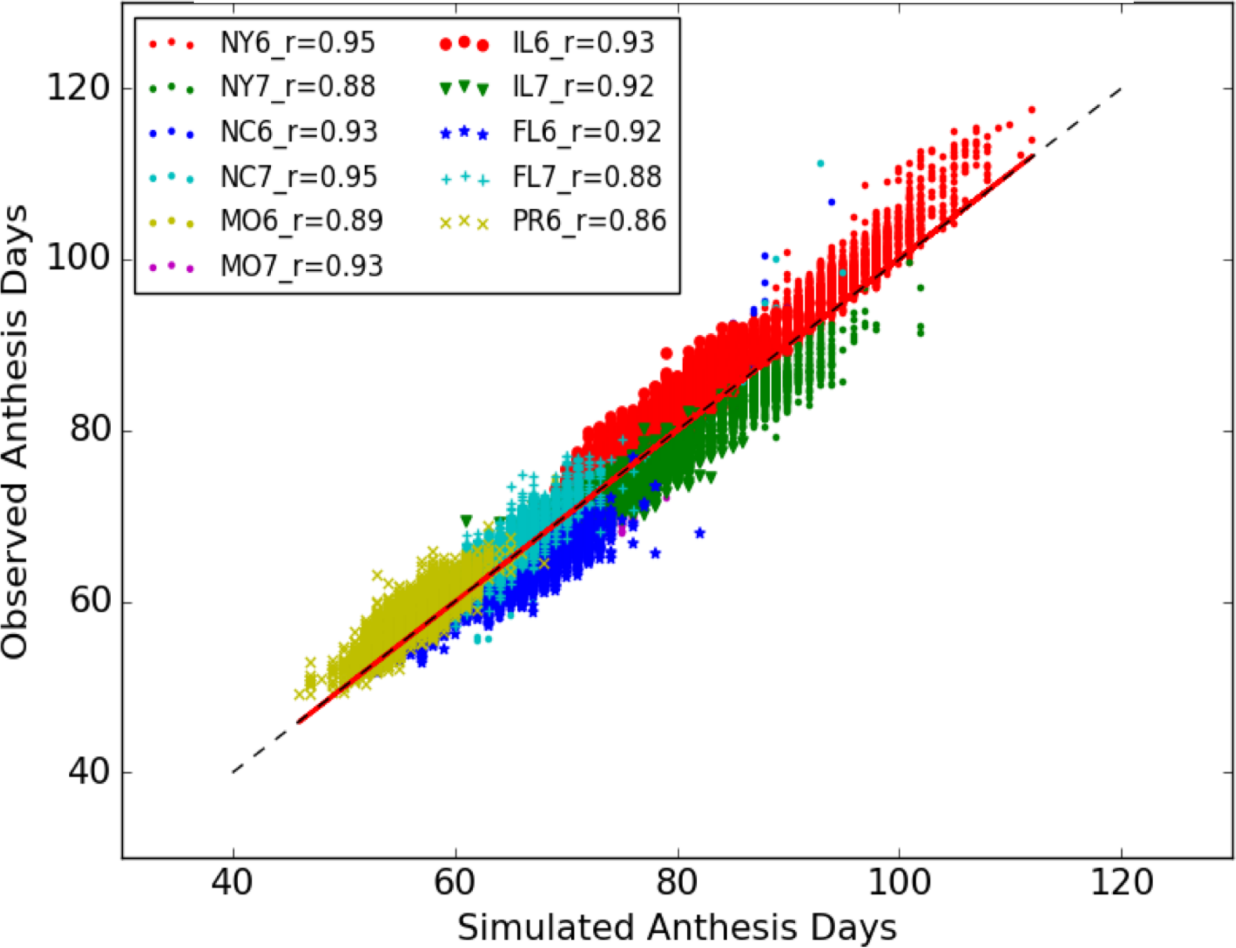
Simulated and observed anthesis days of all 5,266 lines from 11 site-year combinations.

The overall model fit was quite good. In plots of observed vs. simulated days to anthesis for the 49,491 line × 11 site-year combinations (Fig 2), the symbols were concentrated along the identity line with an overall estimated RMSE of 2.39 days. To put this value in context, some other studies have reported RMSEs of 0.91 to 3.2 days [38,39], prediction errors of 6 to 12 days [40]; a mean deviation of 10.1 days [41]; and a standard deviation of 6 days [42]. The detailed scatter plots and RMSE for each site-year are presented in Fig A in S3 File Pearson correlations ($\hat{r}$) were high across site-years and ranged from 0.86 to 0.95, indicating an overall responsiveness of anthesis dates across lines to the range of site-year conditions. The standard deviations of the simulated values and their corresponding observations were 10.336 and 10.639 days, respectively, which, with the overall empirical correlation coefficient of 0.974, accounted for the closeness of the regression of observations vs. simulations to the identity line [43].

### Equifinality

**Fig 3:**
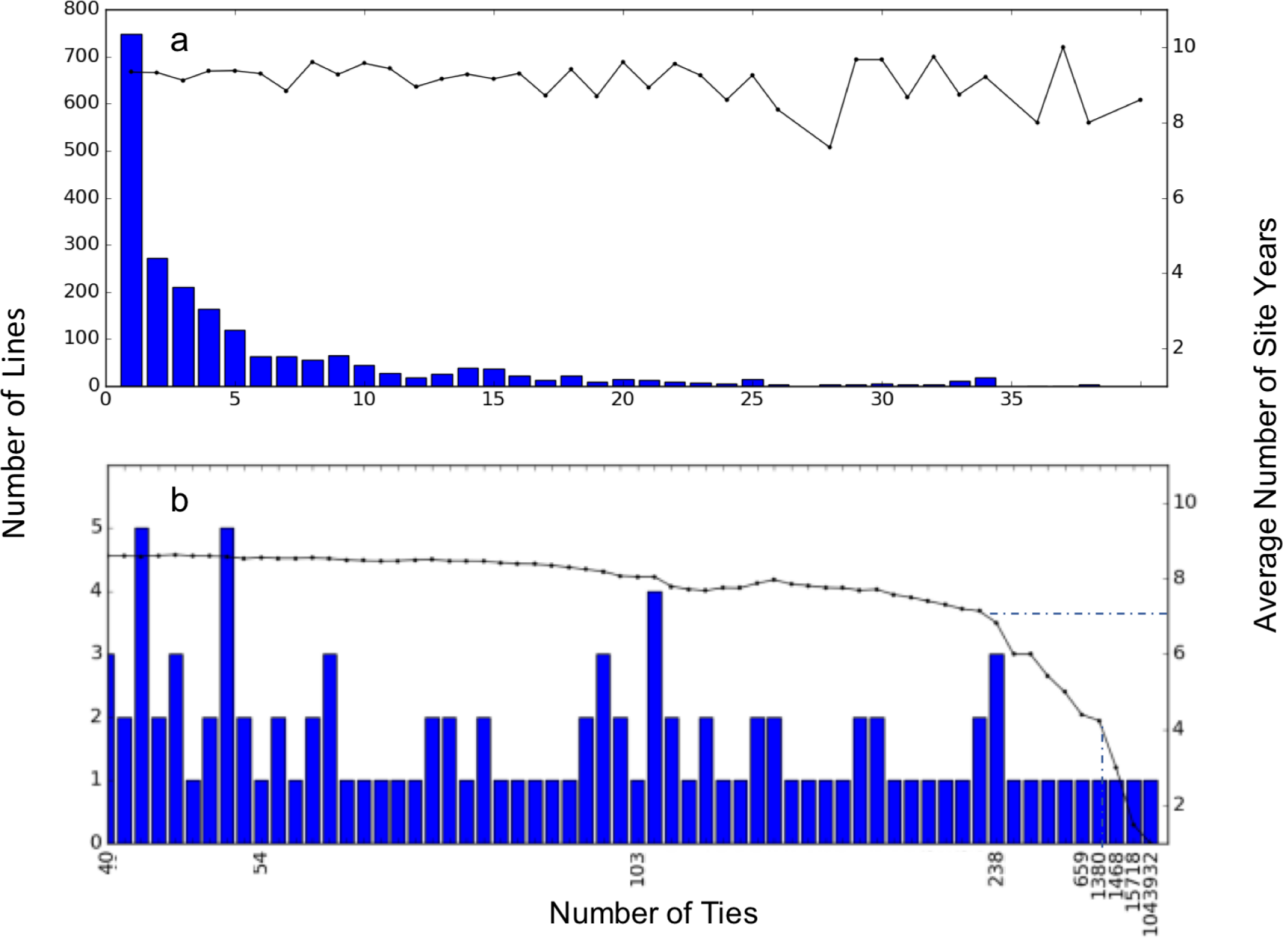
The extent of equifinality (number of ties) for 2,254 lines. (a) 2153 lines with 40 or fewer ties and (b) ties for the remaining 101 lines. The traces (right axes) show the average number of site-years for lines having different numbers of ties.

A total of 2254 lines exhibited equifinality. Of these, 2153 lines had 40 or fewer ties (Fig 3A) and the remaining 101 had from 41 to over 1 million ties (Fig 3B). The number of ties per line (traces in Fig 3A and 3B; right axes) was extreme when there were fewer than seven observations per line (Fig 3B).

**Fig 4:**
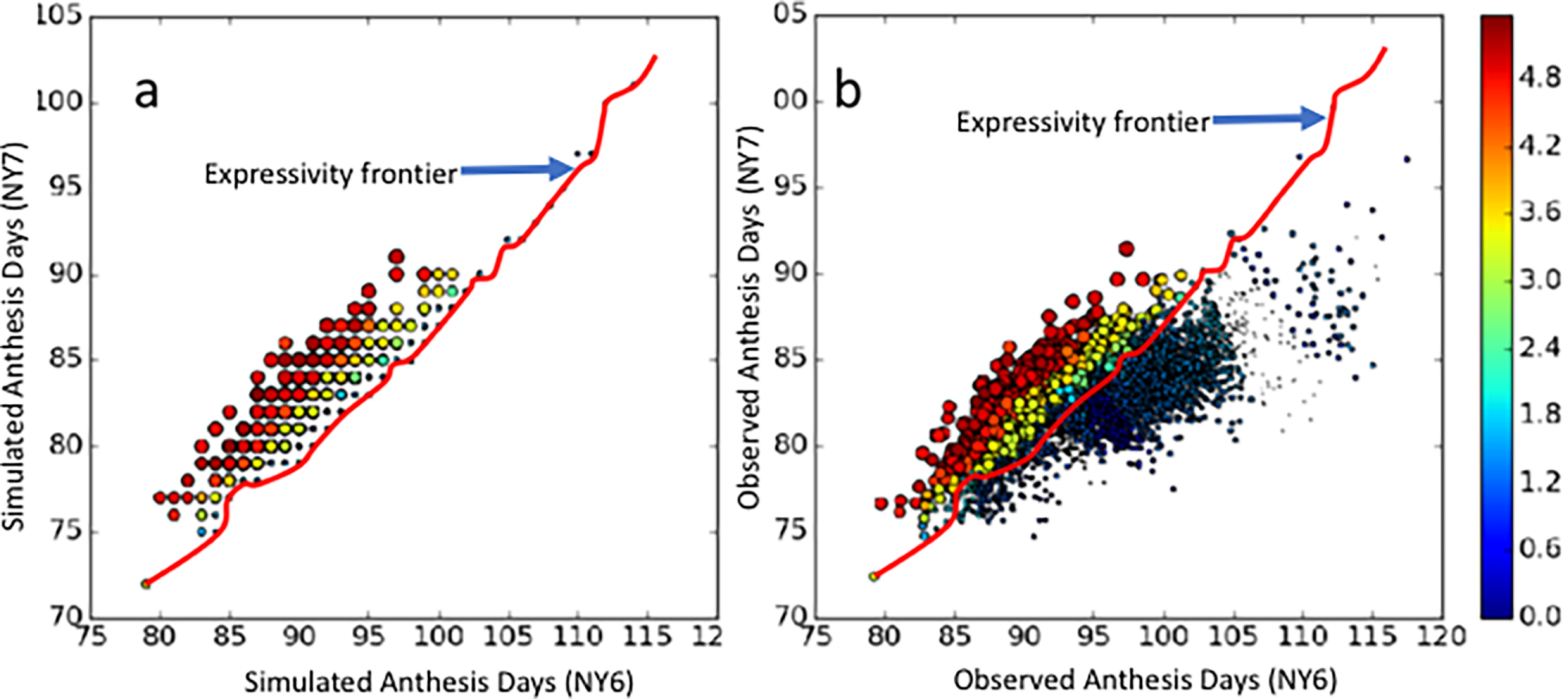
Phenotype space plots. a) simulated and b) observed values of anthesis dates for site-years NY6 and NY7. The red line (copied in both panels) separates observations that the model could vs. could not reproduce. NY6 was cooler than NY7 (Table A in S1 File). The symbol color shows the extent of P1 equifinality on a log_10_ scale.

Anthesis dates for each line common to both NY6 and NY7 are plotted at coordinates corresponding to their paired simulated (Fig 4A) and observed (Fig 4B) values. The seemingly smaller number of data symbols in Fig 4A is due to identical simulated anthesis dates for many lines, leading to overlap in the plot. The symbol colors show the extent of P1 equifinality on a log_10_ scale. The symbol sizes encode the ranges of the equifinal P1 estimates for each line as a percentage of the mean. These vary from 0.36% for the smallest symbols to 65.68% for the largest. The association of redder colors with larger symbols indicates that the ranges of equifinal GSP estimates do, indeed, increase with the extent of equifinality.

When the data were plotted at their observed dates (Fig 4B), the resulting cloud was more dispersed than that of the simulated symbols (Fig 4A), showing the model responses to the environment were less variable than responses of real plants. However, a large number of lines (blue symbols in Fig 4B) had observed anthesis dates that failed to overlap any of the simulated values (Fig 4A). This is indicated by the red line, which was drawn in Fig 4A and then copied exactly into Fig 4B. From here forward, the red line is referred to as the “expressivity frontier”. It differentiates those observations that the model is able to reproduce–above the expressivity frontier–from those below the frontier, which the model cannot simulate. To our knowledge, most previous phenology modeling studies, not only in maize [41] but in other crops as well (e.g., wheat [44], soybean [30], rice [45]), have reported results solely in terms of prediction accuracy without recognizing this second, quite distinct, and unexpected category of model misbehavior. The deleterious effect of this phenomenon on the ability to link ecophysiological models with genetics is examined further in the Discussion. Related details are discussed further in the section on “Model Expressivity”.

### Relations among parameter estimates

**Fig 5:**
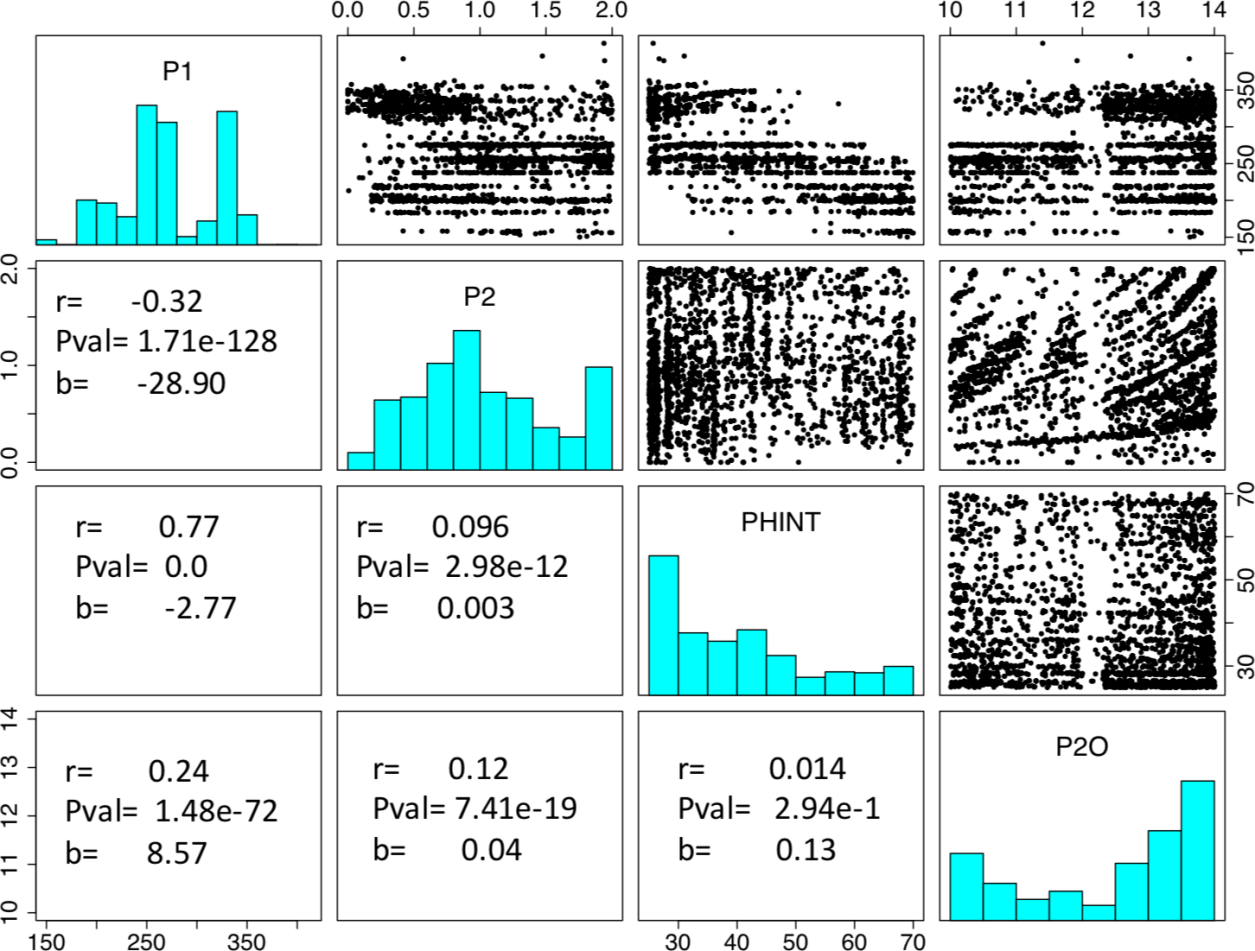
**Empirical distribution of selected GSP estimates (main diagonal), pairwise scatterplots (upper right) and estimated Pearson correlation coefficients, regression coefficients, and *p*-values (lower left).** Each symbol in the scatter plots represents a pair of GSP estimates from a single maize line.

In examining possible relations among parameters, two anomalous features were noted (Fig 5). First, a pronounced banding pattern appeared in all plots except, perhaps, P2O vs. PHINT. Most bands were linear except for those on the scatterplot of P2O and P2, which showed curvature. Second, a vertical gap appeared in all P2O scatterplots. These patterns proved to be symptomatic of serious issues in the estimation process as described further in sections below.

### Model expressivity

**Fig 6:**
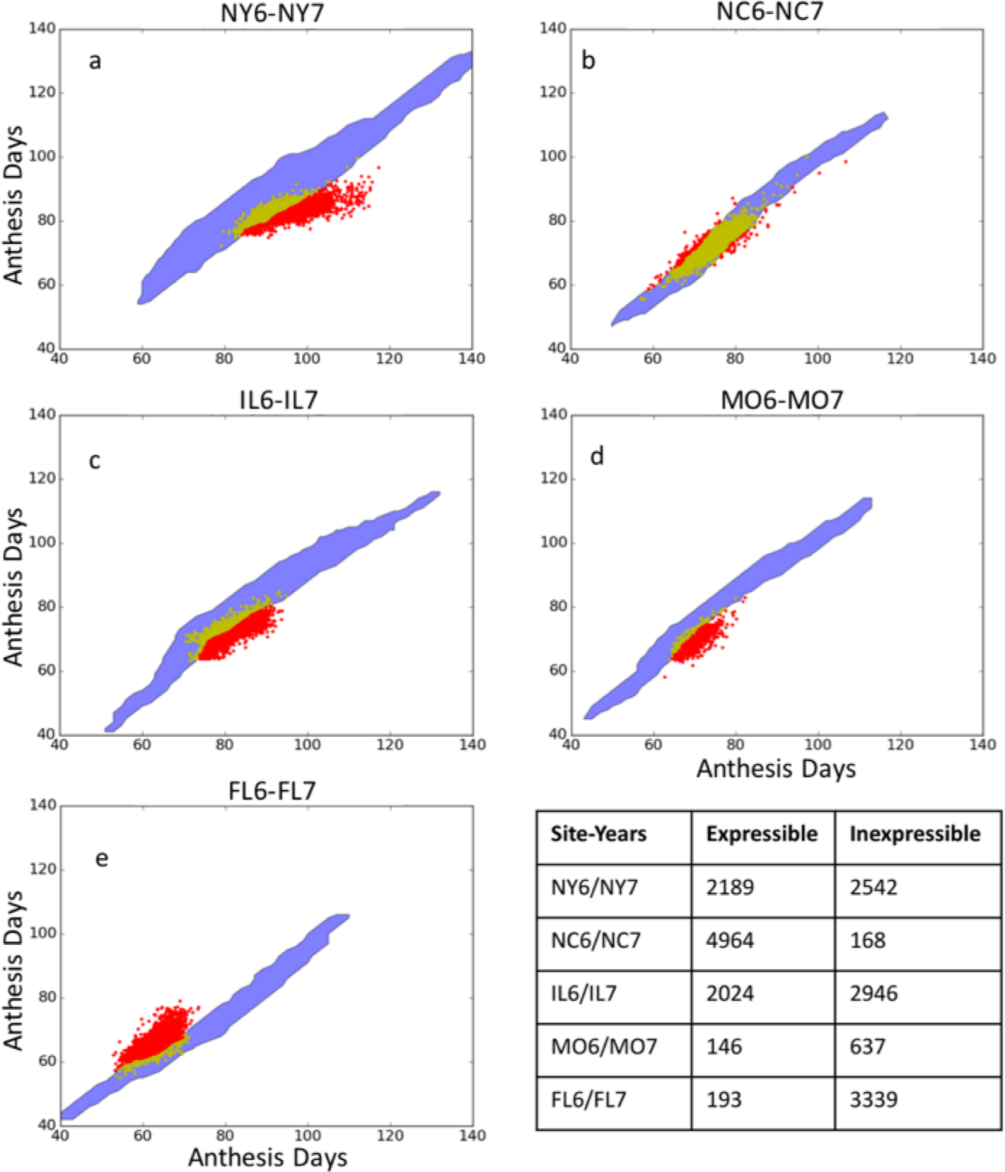
Phenotype space plots for simulated and observed anthesis dates for five site-year pairs. Blue regions outline simulated anthesis date pairs using Sobol database GSP estimates. The data symbols depict expressible (yellow) and inexpressible (red) observed anthesis dates.

To understand the patterns in Fig 4, we explored the “phenotype space” delimited by the observed and simulated anthesis date data for all sites where more than one year of data existed (Fig 6). Except for North Carolina, there were many lines for which no GSP values in the ranges examined allowed the model to reproduce the observed anthesis data. Such observations are hereinafter termed “inexpressible”, and the remaining data are described as “expressible”.

Although the parameter ranges used were in general agreement with prior biological knowledge, the possibility remained that inexpressible observations resulted either because the ranges were too narrow or due to some artifact of the Sobol database method itself (e.g., its discrete character). To evaluate these possibilities in a computationally efficient way, the CERES-Maize anthesis date routine was ported to Python and fit to a single pair of site-years (NY6/NY7) using differential evolution (DE; [46], a well-established, continuous optimization algorithm. Like the Sobol database algorithm, DE allows range limits to be set. Intentionally disregarding prior biological knowledge for the purposes of this test, these limits were specified to be much broader than expectations based on maize biology (Table 3).

**Table 3:** Extended range of parameter values used for DE search.

	P1 (GDD °C)		P2O (h)		P2 (rate)		PHINT (GDD °C)	
Range (Min & Max)	75	600	6	21	0	6	20	100
Percent of Sobol Range	175%		300%		375%		200%	

**Fig 7:**
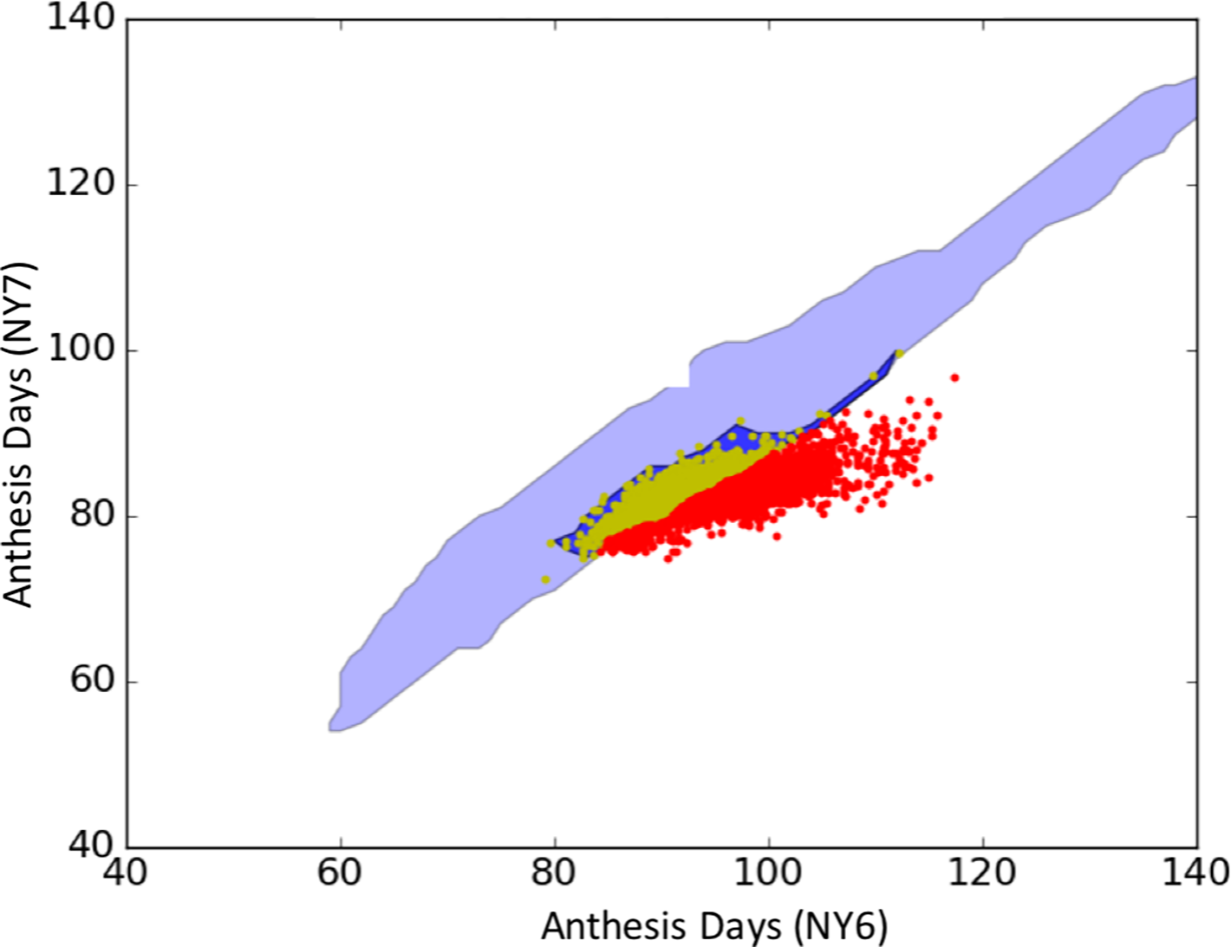
NY6/NY7 anthesis date results from DE (dark blue) and Sobol database (light blue) searches.

Despite this much larger parameter space, the expressivity range of the model was not extended. The results of the DE searches (Fig 7, dark blue) almost exactly reproduce the expressible data (yellow data symbols) but do not extend beyond Sobol database region (light blue) to reach any previously inexpressible observations (red data symbols). This suggests that there might be intrinsic expressivity issues in the model, at least as used in this study, that go beyond the search algorithm used or the parameter space examined.

During the review process, the utility of these extended parameter ranges was questioned. Reviewers also suggested that the NY6/NY7 results might be accounted for by not having optimized the base (Tbase) and optimum temperature parameters (Topt). These parameters control the conversion of daily temperatures to thermal time increments. To explore these twin issues, a six-parameter scan was done that (1) included Tbase and Topt and (2) set the parameter ranges according to values recommended for CERES-Maize in the cultivar and ecotype files provided in DSSAT v4.5 (Table A in S4 File). These ranges are slightly narrower than those in Table 2. The results were that (1) inclusion of Tbase and Topt had no qualitative impact on the results and (2) any quantitative improvements were more than offset by the parameter range narrowing. Specifically, expressivity declined at all sites. The parameter ranges used and the resulting analogs of Figs 5 and 6 are, respectively, shown in Table A and Figs A and B in S4 File

**Fig 8:**
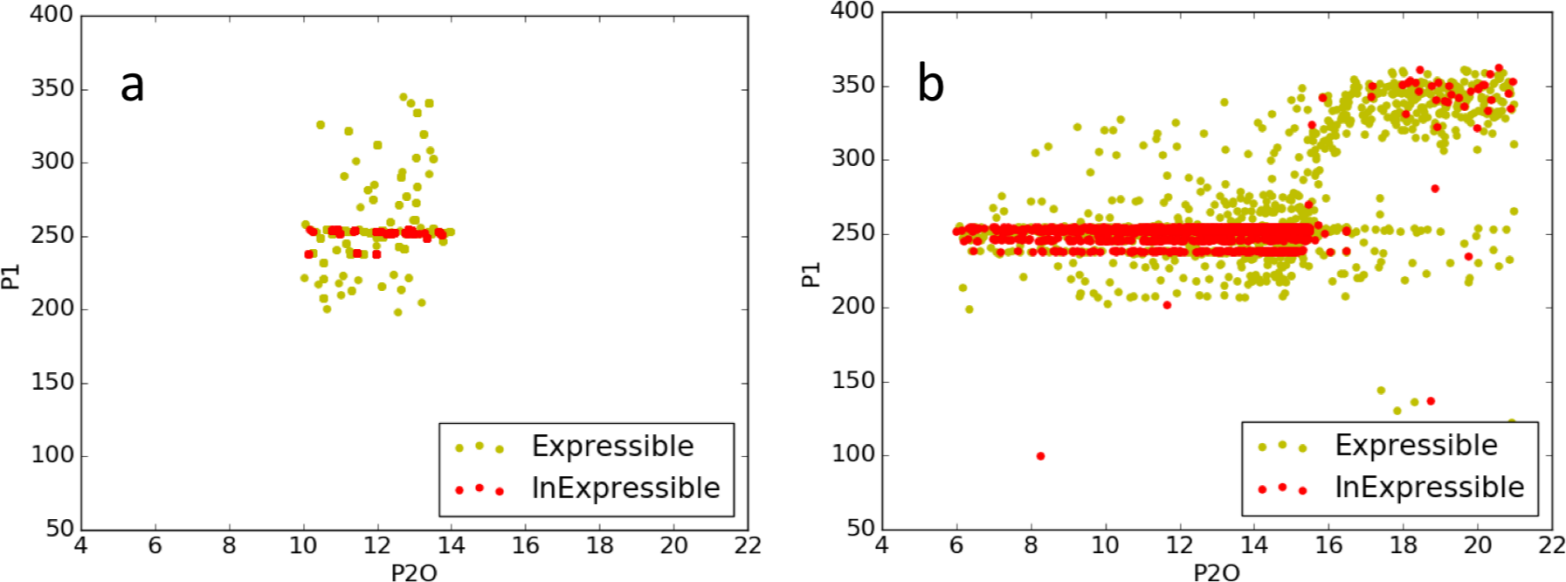
Scatterplot of P1 vs. P2O NY6/NY7. Estimates using (a) Sobol database searches and (b) DE.

A deeper investigation (Fig 8) of the values estimated for expressible (yellow) and inexpressible (red) observations demonstrated a link to the scatterplot banding in Fig 5 for P1 and P2O. In particular, banding was very pronounced near P1 = 250. Tabulation for the Sobol database estimates (Fig 8A) revealed that 68.2% of the lines had P1 estimates ranging from 245–260. Of these, 31.7% (36.5%) of the lines were expressible (inexpressible). Similar proportions were found for the DE estimates (Fig 8B), reinforcing the kindred results of the two search methods. Despite their superficial visual differences, both graphs have 4,731 symbols, the number of lines planted in both NY6 and NY7. The number of apparent symbols is fewer in 8a because of the discrete nature of the Sobol database. This contrasts with 8b due to the fact that DE is a continuous search and the parameter range is wider

**Fig 9:**
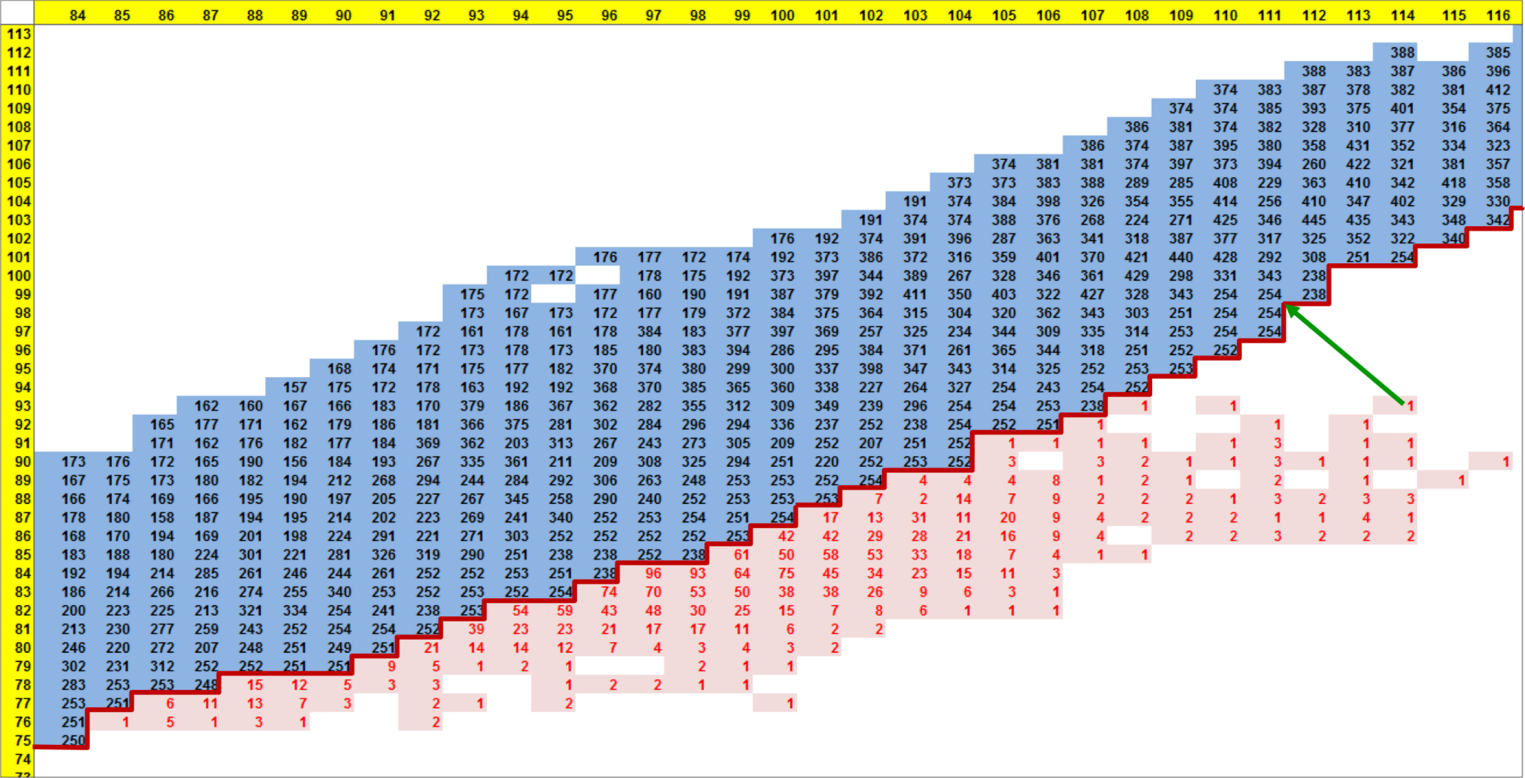
NY6/NY7 phenotype space plot in numeric form. P1 estimates from the database search (black numbers) and the numbers of lines with inexpressible observations (red numbers) arranged in a tableau organized as a phenotype space plot corresponding to the center portion of Fig 8. The inexpressible line at the green arrow would receive a P1 estimate of 254. Horizontal and vertical yellow strips are the anthesis dates for NY6 and NY7, respectively.

Furthermore, a phenotype space graphic in numeric form provided more detail (Fig 9). The black numbers in the blue region are the first-best-found P1 values that generate the corresponding row and column anthesis date combinations. Note that these tend to be close to 250 along and near the expressivity frontier. The red values are the numbers of lines whose anthesis date combinations were not expressible by the model. The RMSE for inexpressible observations was minimized by assigning the GSP values associated with the closest achievable dates, namely the nearest point on the expressivity frontier (e.g. the green arrow). Thus, all lines with inexpressible dates received P1 values of ca. 250, as did lines with expressible observations that fell close to the frontier. This phenomenon accounts for the banding in Figs 5 and 8.

### P2O gap

**Fig 10:**
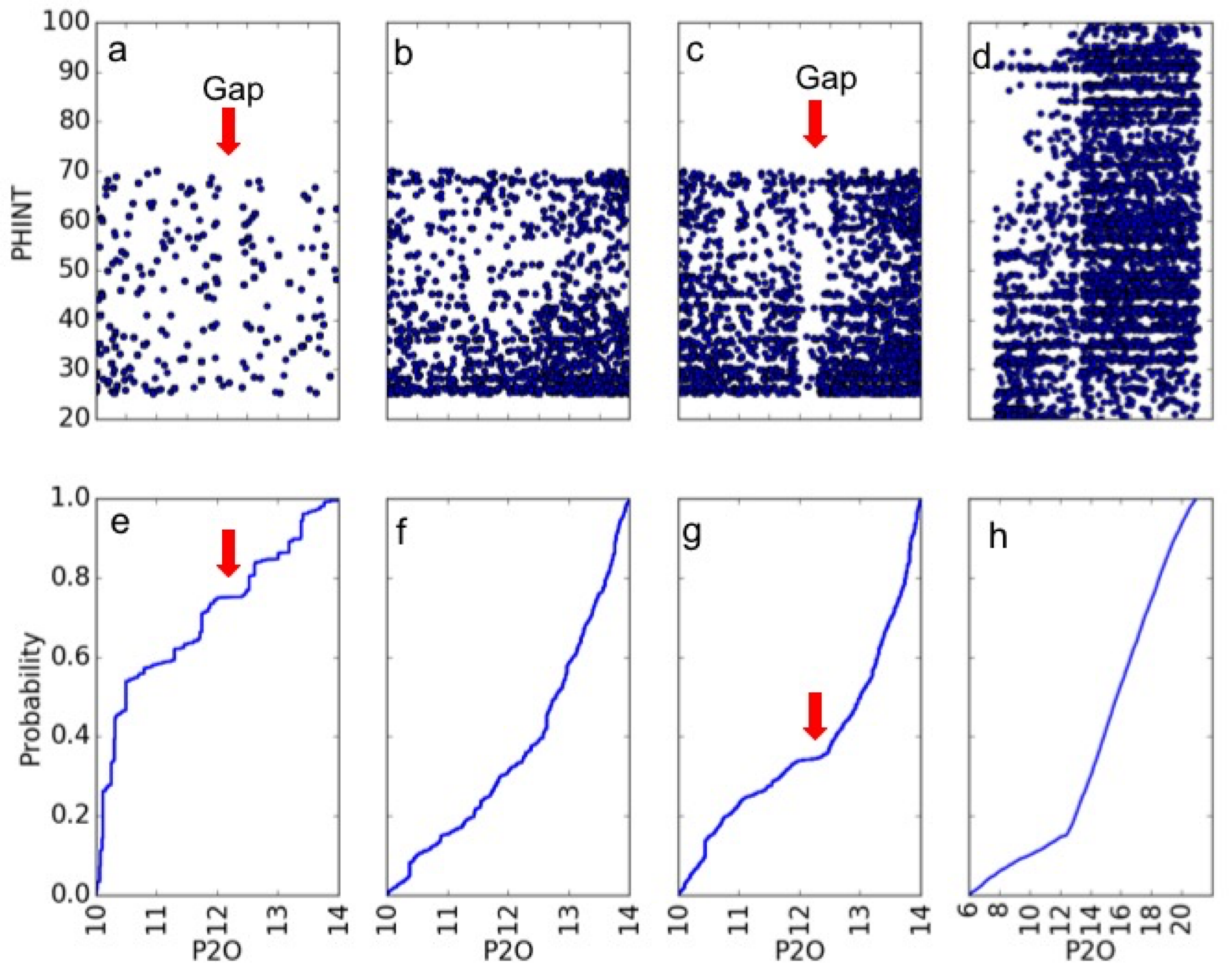
**P2O and PHINT scatter plots (top row) and P2O cumulative density functions (bottom row).** Parameter estimated by the database method using (a, e) 3 southern site-years, (b, f) 8 northern site-years, and (c, g) all site-years; and (d, h) the same as (a, e) but obtained via DE.

Of the 11 site years, three (FL6, FL7, and PR6) had decreasing day lengths during Stage 2, all of which were less that ca. 11.5 h. P2O estimates based on these site-years showed a gap (Fig 10A and 10E). In contrast, the other eight site years all had Stage 2 photoperiods longer than the maximum allowed in the Sobol database search (14 h). P2O estimates obtained using these data exhibited no gap (Fig 10B and 10F). When estimates were computed using any data from the three southern site-years, a gap resulted, in particular as seen in the combination of all 11 site-years (Fig 10C and 10G).

An optimizer will find any way that it can to reproduce the observations, i.e. minimize RMSE. In this case the “P2O gap” results from an interaction between the model’s equations for anthesis dates and the range of parameter values allowed. For any given value of P1, the predicted anthesis date is determined by the combination of P2O, P2, and PHINT values. In southern states with short photoperiods, there were two cases to be considered–lines with longer vegetative periods and those with shorter ones. In the former case, the optimizer would select a P2O that was much shorter than the actual (already short) day lengths. Then it can achieve the needed delays by selecting combinations of P2 and PHINT to create a best match with the dates observed across all sites. For lines with shorter vegetative periods, the optimizer selected P2O values greater than the actual day length so that Stage 2 only lasted for four days. Again, however, the needed intervals were obtained by adjusting PHINT. Because of equifinality, all values of P2O in excess of the observed photoperiod were equally workable in the latter case. Similarly, there were multiple workable combinations of P2O and P2 in the former case. The result was a P2O gap bracketing the actual photoperiods at each of the southern sites.

In the case of northern sites, the actual photoperiods exceeded the 14-hr limit put on the Sobol database. Thus, in the north, all lines were analogous to short vegetative lines in the south. In the effective absence of two categories, no band was seen in the north. When the P2O limit was extended in the DE search, all southern cases with gaps were rendered equivalent to the northern sites and the gaps disappeared (Fig 10D and 10H).

### Tests for stability of GSP estimates

The effect of including or excluding the effect of different subsets of site-years on each GSP estimates (Eqn. 2) was hugely significant (chi-square *p*-values, Table 4). The AIC and BIC values for all GSP’s were considerably smaller for models that included the random effect of site-year subsets, *β*_*e*_, therefore also suggesting non-negligible variability. To illustrate the size of the site-year subset effects, an Index of Variability (IoV) was calculated as the standard deviation of the *β*_*e*_ effect, normalized by the grand mean (the intercept ${\hat{\mu }}_{\rho }$ in Eq 2), and expressed as a percentage. The percentage of the total GSP variance ($\sigma_{e}^{2}+\sigma_{l}^{2}+\sigma_{r}^{2}$) attributable to site-year subsets was also calculated. Both descriptors indicated substantial variability between site-year sets, with indexes of variability ranging from 5.9% for P2O to 33.6% for P2 and over 20% of the total variance related to site-year sets for all GSP’s. All of these statistics demonstrate that the GSP’s based on this model structure are not, in fact, genotype-specific despite the goodness-of-fit displayed in Fig 2. This result is understandable given the range of artifacts due to equifinality and model expressivity issues identified above (Figs 4–10) along with the unevenness of their distribution across site-year x line combinations (Table 4). The possible causes and implications of these finding are discussed next.

**Table 4:** Estimated log likelihood, fit statistics, selected summary measures, and a likelihood ratio test for competing statistical models fitted on GSP estimates with and without the random effect of site-year subset, based on GSP estimates for the base group (N = 60,834).

GSP	Log likelihood w/o (top) and w/ (bot) a site-year set effect^a^	AIC w/o (top) and w/ (bot) a site year set effect^b^	BIC w/o (top) and w/ (bot) a site-year set effect^b^	GSP Grand Mean ${\hat{\mu }}_{\rho }$	Index of Variablility^c^ (IoV; %) $\frac{\sigma_{e}}{{\hat{\mu }}_{\rho }}$	Variance pcts. for site-year sets^c^ $\sigma_{e}^{2}/\sigma_{tot}^{2}$	*χ*^2^ test statistic	*χ*^2^ *p*-value^d^ (df = 0.5)
P1	-338046	676098	676125	264.625	12.30	34.38	30714	10^−13334^
	-322689	645386	645422					
P2	-46154	92313	92340	1.037	33.55	33.92	41833	10^−18163^
	-25237	50482	50518					
P2O	-105304	210614	210642	12.2440	5.88	27.83	19894	10^−8635^
	-95357	190723	190759					
PHINT	-254875	509756	509783	44.167	15.44	22.62	15943	10^−6919^
	-246903	493815	493851					

^a^ Larger is better

^b^ Smaller is better

^c^ *σ*_*e*_ is the site-year subset std

^d^ Chernoff upper bound on Chi-squared cum. dist. function.

## Discussion

Since their inception, ecophysiological models have been evaluated in terms of predictive ability, which is often superb [47]. In such work, ECM parameters were considered to be *inputs* whose genesis was secondary as long as the model outputs proved useful. However, perceived needs, desiderata, and requirements escalate as technologies evolve. It is now expected that the model inputs, themselves, be the accurate outputs of processes at the genetic level that can be modeled by genomic prediction. It is not surprising, therefore, that modeling technologies that were adequate for past applications now require improvement.

Usually experimental GSP measurement requires intricate and/or intensive protocols. GSP’s are also rather numerous in modern ECMs. In combination these factors make their direct determination infeasible for more than a few lines. This mandates indirect inference of GSP’s via model inversion [48]. There are, however, multiple ways that inverse studies can go awry that can affect the usability of the end results. A non-exhaustive list of these includes (1) an inappropriate objective function for measuring goodness-of-fit, (2) inadequate sampling of the parameter space, (3) errors in the observed phenotype data, (4) errors in the model input data, and (5) structural issues and errors in model representation of biological/environmental process interactions.

Relative to the first point, this study employed RMSE as the objective function, making the searches congruent to the virtually universal nonlinear least squares. The second potential pitfall, parameter space sampling, was explicitly addressed in three ways. First, a parameter range was used that is far wider than what might be deemed biologically reasonable (but see the discussion of P2O below). Secondly, the Sobol database approach guaranteed that the range was sampled at a uniform density limited only by the amount of computing power available. Conventional search algorithms, even global optimizers, can be inferior in this regard, especially when objective functions have highly deceptive goodness-of-fit landscapes [49]. Third and finally, the achieved sampling density was maximized by the use of supercomputing resources.

Additionally, many traditional optimization algorithms are like DE in that they only produce single point estimates [46,50,51] and thus lack any ability to assess equifinality or model expressivity. This gives the Sobol database used herein a clear superiority in that it can evaluate the properties of both the parameter and phenotype spaces. Moreover, when used with parameter ranges that exceed those ascertained from prior knowledge, Sobol database methods reveal the upper, structural limits on model expressivity. The degree of expressivity shown when ranges are set by prior knowledge can easily depend on which “prior knowledge” is used (e.g. Fig 6 vs. Fig A in S4 File). A parameter space scan using an extended range establishes a benchmark against which such variation can be interpreted.

With respect to trait scoring errors, anthesis date phenotyping is extremely common and, as described in the Methods section, standard sampling methods were used. While site-specific observer effects cannot be discounted as being present, these same data sets have been successfully used in a variety of other studies [4,5]. Even if adverse observer effects were to be documented at some later time, they would only reinforce the central point of this paper, namely that refined protocols for GSP estimation are required.

Relatedly, an examination of the weather data inputs (germane to point 4) was instructive. As shown in Table A in S1 File, when compared to a gridded data set, there were small, site-dependent, near-constant, inter-annual offsets (i.e., biases) and/or swings (i.e., variation). Moreover, these had variably-sized effects on predicted anthesis dates (Fig C in S1 File). Of course, it is not possible to tell which is more accurate, the gridded data or the local observations. Although soil and terrain data were not used in this study, their notorious spatial variability would only exacerbate whatever issues are attributable to weather data herein. Indeed, at least one GSP estimation study has documented improved prediction stability with increases in the amounts of soil data used [25]. More on these issues will be stated below along with discussion related to model structure (point 5).

Inadequate expressivity is more damaging than equifinality because it is unlikely to be alleviated by optimization constraints in the form of more data or more data types. The only solution for a lack of model expressivity is to develop models that better represent underlying physiological processes. For example, although CERES-Maize includes equations for estimating emergence dates, the corresponding GSP’s for this component were left at default values, because emergence data were neither in the published dataset and nor the field trial reports (E. Buckler, personal communication). This effectively removed seedling emergence as a means to distinguish between lines. Extrapolating this example, one can readily imagine models that omit other critical processes altogether.

Adding new processes to such models will likely require estimating more GSP’s, similar to what would have been necessary to include emergence date data in this study. However, this will increase equifinality. Large numbers of equifinal points (e.g., a million ties in some cases) is a mathematical issue resulting from model structure (an aspect of point 5, above) and data. Stated graphically, model revisions that move the expressivity frontier down and rightward in Fig 4B will make more symbols redder and larger. The overall solution is to first increase model expressivity and then to include more observations of more model variables to reduce equifinality.

Current research and development efforts aimed at high throughput phenotyping (HTP) technology will be helpful in adding new data types. For example, if one assumes that TOLN = SUMDTT/(PHINT×0.5) +5 is the correct way to model the number of leaves at anthesis, then HTP data on total leaf number would allow optimizers to particularly favor PHINT trial estimates that approximated 2×SUMDTT/(TOLN-5).

In real world situations, equifinality concerns not only model parameters, but also gene action. However, when gene-level equifinality occurs (i.e., multiple pathways produce the same phenotype), one would expect the mapping step to reveal all active, contributory genes. When alternative sets of genes are stably present and act toward similar ends across environments then, *ipso facto*, their markers will have strong associations with the GSP values. Thus, genetic equifinality would be detected during the GSP mapping phase.

Alternatively, another approach to reduce equifinality is to simplify models. Simpler models would have fewer GSP’s and fewer indirect mathematical pathways through which changes in one parameter could be exactly offset by changes in others. Of course, fewer GSP’s, along with possible reductions in the range of processes modeled might limit model plasticity to the detriment of expressivity. Whatever path is taken, the Sobol scheme herein can be used to assess trade-offs between model equifinality and expressivity, thus providing a valuable tool in facilitating the linkage of more fundamental biological traits with their underlying genetics.

However, forging links between traits and genetics requires parameter stability. Instability can occur when: (1) undiscovered equifinality is present, and the solutions found depend on low-level algorithmic idiosyncrasies of the optimizer; (2) a stable answer exists but the optimizer is insufficiently skilled to find it; (3) a stable and possibly even unique answer exists within the skill level of the optimizer to find, however, because of a large number of parameters, the values obtained reflect noise signals that differ between environments; or (4) the model incompletely or incorrectly disentangles G × E.

Explanation (1) is unlikely in this study, first because rampant equifinality was, in fact, discovered and ties were explicitly excluded from the evaluation of instability and, second, two different optimizers (Sobol and DE) performed similarly. Explanation (2)–unskilled optimization–also seems unlikely to be present given the small RMSE values achieved. Explanations (3) and (4) are interrelated in that they are both additional examples of model structural issues, which was point (5) in the above list of GSP estimation frailties.

In this study, there were detectable systematic differences in the weather data collected in different site-years (Fig A in S1 File). Moreover, although small (Table A in S1 File), these differences had a measurable effect on anthesis date predictions (Fig C in S1 File). However, the use of very large data sets confers an extraordinary and, perhaps, excessive power to detect GSP site-year dependencies (Table 4). For example, a visual comparison of Fig C in S1 File with the NY and FL panels in Fig 6 shows that the effects due to weather instabilities are insufficient to compensate for lack of expressivity. Thus, it seems likely that there are remaining G x E disentanglement issues in this model.

To the extent that the statistical instability test is deemed overly sensitive, the IoV might be a better index for practical interpretation. Even so, a clear implication is that field researchers must seek methods of abiotic measurement that better characterize the actual environments experienced by the plants. For example, by combining high temporal and spatial resolution canopy temperature data from UAV-mounted sensors with an ECM that simulates crop development responses to canopy temperature, external measurements of some environmental variables (e.g., air temperature) could, perhaps, be foregone.

Whatever is done, of course, one cannot accurately estimate the controlling parameters without collecting data in settings wherein the relevant processes operate differentially. Another problem can arise from adverse interactions between model structure (point 5 yet again) and the specification of prior knowledge. This is clear from a comparison of the P2O results for Sobol database and DE searches. In the former, the estimates were unrealistically compressed into two restricted ranges separated by a gap (Fig 10A, 10C, 10E and 10G). During the second run, however, because of equifinality in combination with wider permitted ranges, the optimizer (DE) found a different way to “explain” the observed anthesis dates. Specifically, it shifted the most important explanatory variables from P2O, P2, and PHINT to PHINT alone. This eliminated the gap found in the first search but spread the P2O estimates out until they attained values considered biologically unrealistic (Fig 10D and 10H). This forcefully makes the point that unexpected, highly counterintuitive, and even counterfactual interactions can occur during estimation. Such artifacts might not have been observed before because previous studies (e.g. [41]) have not explored the parameter space with methods able to reveal them.

The debilitating influence of all of the behaviors seen herein on attempts to link parameter values to genes is, unfortunately, quite obvious.

An additional concern with quality of the data was that only 539 out of 5266 lines had anthesis date observations for all plantings, and, where anthesis data were lacking, no information was given as to whether the field plot died prior to expected anthesis date or failed to reach anthesis, presumably due to high photoperiod sensitivity. Besides documenting a need for more complete plot-level data, this imbalance in representation of lines across environments suggests that some more global notion of balance needs to be established and applied for use in ECM inversion. However, given the expense of such large-scale trials and the multiple purposes they serve, “balance” cannot mean “orthogonality” with all lines planted at all sites. There is a large literature on methods for optimizing experimental designs [52–54]. Perhaps such methods should be applied at levels higher than the single field trial with the needs of GSP estimation being a specific criterion receiving consideration.

## Conclusions

The anthesis date component of the CERES-Maize model was fitted with data from 5266 maize lines including the maize NAM population. Despite the model’s high predictive ability, issues of expressivity, equifinality and instability were identified. Although analysis of GSP’s as crop traits still seems highly promising, the problems noted with CERES-Maize simulations of anthesis date were severe enough to preclude use of its estimated GSP values in mapping analyses. Model inversion using the Sobol database approach proved especially useful because, unlike other optimizers that find single point estimates of GSP’s, this algorithm revealed both the extent of equifinality and the boundaries of the expressible phenotype region. It should be employed more broadly, for example, to additional models of maize phenology [41] and, beyond this, to complex traits in other crop models.

The constraining issues can be summarized as falling mainly into three categories. The first arises in situations where the model is unable to express the observed data for some lines, even by a relatively few number of days. In this circumstance, a line is assigned the GSP associated with the nearest point on model’s expressivity frontier. The result is that many, even a majority, of lines are assigned the same GSP values independent of their genetics. The second issue arises when the model can reproduce the data but there are many combinations of GSP values that predict equally well, i.e. equifinality. When equifinality exists, there is no principled way to assign the line a genetically relevant value. The third issue, which can arise in either equifinal or inexpressive situations, is when GSP estimates are unstable, i.e., they vary depending on the set of environments used to determine them. In this case, simulation outputs will be suspect when the model is applied to environments not used in estimation.

The importance of these issues cannot be overstated. Community interest in the GSP-fitting-and-mapping paradigm is high as shown by the heavy citation rates for the seminal papers in this area. For example, as of January 2018, the classic [7] paper had been cited 304 times and those publications, themselves, had been cited by 9,713 others (Source: Google Scholar). Indeed, it is quite unclear that there is an alternative approach for linking genotypes to phenotypes in situations involving non-constant environments and interacting, nonlinear biological processes. However, without the ability to obtain stable and unambiguous GSP estimates that fully reproduce the data’s observational range, the paradigm breaks down. Therefore, it is mandatory that the GSP estimation issues raised herein be addressed and resolved.

Of course, one cannot fix problems one cannot first detect. Doing so will require more and better data, but also improved metadata. For example, there is a need to consider better ways to quantify the abiotic conditions actually experienced by the plants as well as the protocols and quality of that data. On the biotic side, we need more crop development and status data, as discussed in the emergence date example above. Current burgeoning research on high throughput phenotyping may help meet critical needs in this area by expanding not only the range of traits that can be quantified but also increasing their temporal frequencies and reducing the errors (especially observer effects) inherent in manual measurement.

Additionally, more than 11 site-years are needed for this type of work. The CERES-Maize GSP’s studied here had large values for the Index of Variability because only 11 site-years of data were used. Therefore, it seems highly unlikely that the values obtained will generalize. Even if the IoV values had been much smaller, it is hard to believe that 11 plantings adequately capture the environmental variability of US corn production. High throughput phenotyping will also help to meet this need.

However, all of these improvements will increase computational loads despite the efficiencies of the Sobol database method when used for large numbers of lines. Therefore, strong consideration should be given to disaggregating comprehensive models into separate modules that can be studied independently at much lower computational cost. (This was done here when the anthesis submodel was ported to Python to study an expanded parameter space.) A good long-term strategy would be to program future models in a manner that supports single-module testing at the source code level.

In conclusion, there is no doubt as to the importance of the ability to predict the behaviors of novel genotypes in novel environments while crosses are still in the planning stage. Indeed, this is precisely the genotype-to-phenotype problem, which has been declared by the National Research Council to be a top-priority goal for applied biology [1]. So, these impediments need to be overcome. With methods like the ones advanced here for detecting adverse model behaviors under estimation; emerging technologies for collecting ever larger and higher quality data sets; research that is probing ever more deeply into the plant biological processes and their controls; and despite the huge amount of work to be done, there is no reason to believe that we will not be successful.

## Supporting information

S1 File Analysis of the quality of daily weather data. (DOCX)

S2 File Ties. (DOCX)

S3 File Observed vs Simulated scatter plot for each site year. (DOCX)

S4 File Six parameter scan with cardinal temperatures. (DOCX)
