# Supplementary material for: Estimating parametric phenotypes that determine anthesis date in Zea mays: Challenges in combining ecophysiological models with genetics
Source: PLoS One. 2018 Apr 19;13(4):e0195841. doi: 10.1371/journal.pone.0195841 (PMC5909614; doi:10.1371/journal.pone.0195841)
Supplement: S1 File — (DOCX) [file pone.0195841.s001.docx]

Given the well-documented problems with data from individual weather stations [1,2], a possible concern with our results is that the differences in modeled anthesis dates for 2006 and 2007 might have resulted from artifacts related to weather data. These might arise due shifts in instrument sensitivities, changes in station exposure or similar factors. Because the data were provided by the authors of a genetic analyses for photoperiod response [3], we did not have access to detailed information on weather station management or siting relative to the actual experiments.

To assess possible bias for 2006 vs. 2007 data, we compared the daily station data with gridded high-resolution 2.5 arc minutes (ca. 4 km) surface meteorological data prepared by [4], considering data from planting to 100 days after planting for each site-year combination. The gridded data were modeled using a hybrid approach intended to reduce bias of individual stations by combining two sources providing temporally rich and very high spatial resolution data.

Fig A compares the time trends of station vs. grid data for daily mean temperatures. The trends for station and grid data are similar but for individual site-year combinations, differences between the two data sources approached 5 °C on single days. Expressing the data as the difference of station and grid data made these deviations more apparent (Fig B). Mean differences for the 101-day intervals ranged from -0.6 to 0.4 °C.

The foremost concern for our analyses was whether, for a single site, there was evidence that the station data and gridded data differed in a way that might account for the large year-to-year differences in the relationships between predicted vs. observed anthesis dates (Fig B). The magnitudes of the interannual swings range from 0.1 °C at Aurora, NY, to 0.8 °C at Homestead FL (Fig B and Table A). These sites were analyzed in detail as follows.

Temperature effects on anthesis dates in CERES-Maize are modeled in terms of degree-days above a base temperature parameter (Tbase) that was set to 8 °C in all estimation runs. As temperature integrals, degree-days are closely related to temperature averages. To assess simply the impacts of the apparent temperature swings seen, CERES-Maize runs were done wherein the Tbase parameter was adjusted so that degree-days accumulated at the same rate they would have under the gridded data. This was done for all lines present in both NY plantings and both FL plantings. All other parameters were left at their optimal settings as determined by the Sobol database search.

The results are shown in Fig C While there are systematic differences, these are on the order of plus or minus one day for ca. 85, 98 and 78% for FL6, FL7 and NY7 sites respectively. In comparison the average days to anthesis are 95.19, 82.79, 66.51, 63.37 for NY6/7 and FL6/7 respectively. The implications of these results are elaborated on in the main text Discussion section.

Table A. Comparisons of station vs. grid cell data for daily mean temperature up to 100 days after planting for the six sites where trials were conducted. Station data are as used in the simulations, and grid data are values from the corresponding 2.5 arc minutes grid cell locations from modeled surface meteorological data of [4].

|  |  |  |  |  | Daily mean temperature (°C) | | | | | | | |
| --- | --- | --- | --- | --- | --- | --- | --- | --- | --- | --- | --- | --- |
|  |  |  |  |  | 2006 |  |  |  | 2007 |  |  | |
|  |  |  |  |  | Mean |  |  |  | Mean |  |  | |
| Site | Latitude | Longitude | Elevation (m) | | Station | Grid | Difference | | Station | Grid | Difference | |
| Homestead, FL | 25.510 | -80.498 | 2 |  | 22.96 | 23.41 | -0.45 |  | 23.09 | 22.82 | 0.27 |  |
| Urbana, IL | 40.080 | -88.230 | 220 |  | 22.11 | 21.83 | 0.28 |  | 23.50 | 23.08 | 0.42 |  |
| Columbia, MO | 38.895 | -92.201 | 270 |  | 24.45 | 25.00 | -0.55 |  | 24.04 | 24.55 | -0.51 |  |
| Clayton, NC | 35.670 | -78.493 | 105 |  | 23.66 | 23.22 | 0.44 |  | 23.72 | 23.36 | 0.36 |  |
| Aurora, NY | 42.730 | -76.660 | 250 |  | 19.58 | 19.55 | 0.03 |  | 19.90 | 19.81 | 0.09 |  |
| Ponce, PR | 18.001 | -66.510 | 8 |  | 26.5 | (na)^*^ | (na) |  | (na) | (na) | (na) | |

* (na) = Not available. The gridded dataset was only for the continental US, and no trial was conducted at Ponce, PR in 2007.


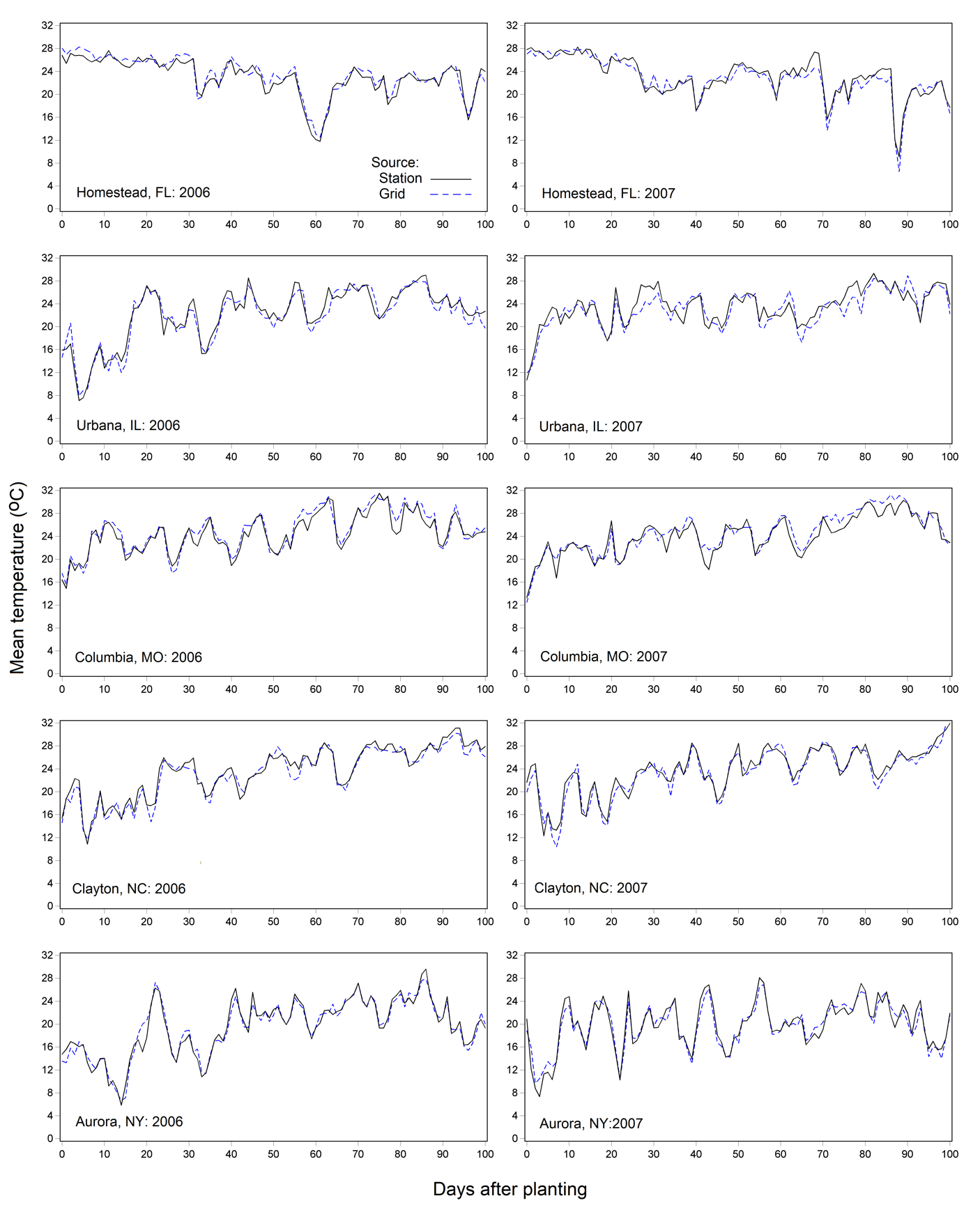


Fig A. Time trends for daily mean temperature up to 100 days post planting for the five sites where trials were conducted both years. Station data were used in the simulations, and grid data are values from the corresponding 2.5 arc minutes gridded surface meteorological data of [4].


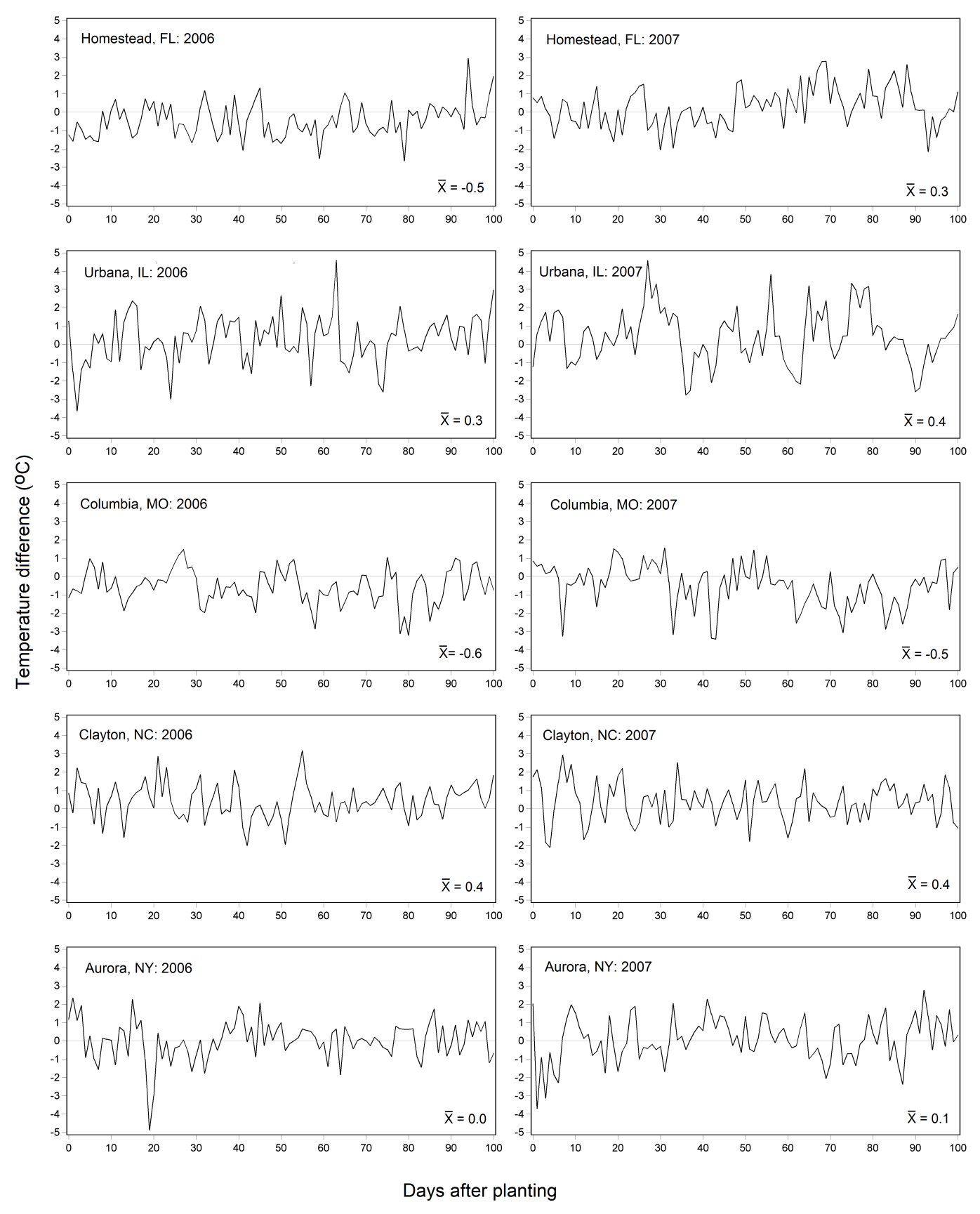


Fig B. Difference in time trends for daily mean temperature as shown in Fig A (calculated as station less grid values) for the five sites where trials were conducted both years. Station data were used in the simulations, and grid data are values from the corresponding 2.5 arc minutes gridded surface meteorological data of [4].

**
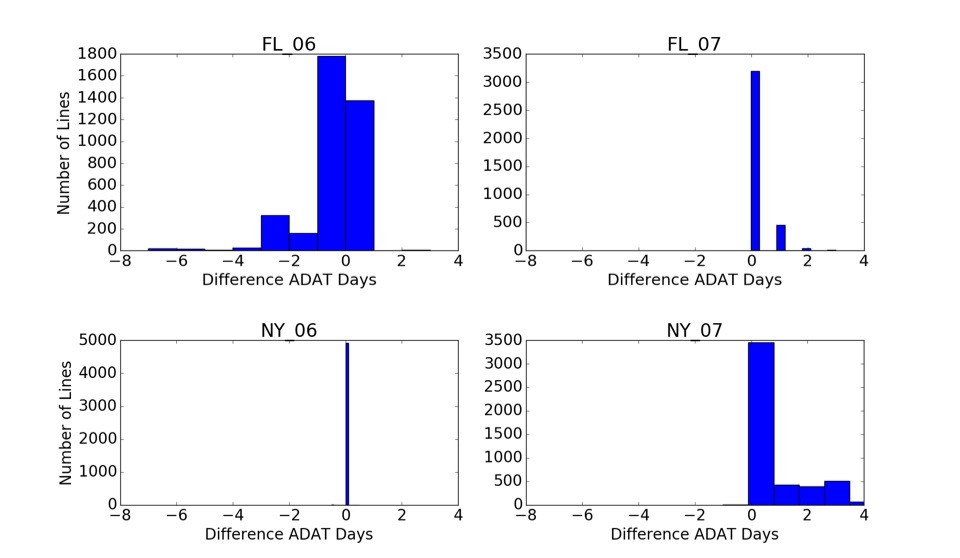
**

Fig C. Histogram of difference in simulated anthesis days using study’s site weather station and gridded weather station.

**References:**

1. Fall S, Watts A, Nielsen-Gammon J, Jones E, Niyogi D, Christy JR, et al. Analysis of the impacts of station exposure on the US Historical Climatology Network temperatures and temperature trends. J Geophys Res Atmospheres. 2011;116. Available: http://onlinelibrary.wiley.com/doi/10.1029/2010JD015146/full

2. Pielke RA, Davey CA, Niyogi D, Fall S, Steinweg‐Woods J, Hubbard K, et al. Unresolved issues with the assessment of multidecadal global land surface temperature trends. J Geophys Res Atmospheres. 2007;112. doi:10.1029/2006JD008229

3. Hung H-Y, Shannon LM, Tian F, Bradbury PJ, Chen C, Flint-Garcia SA, et al. ZmCCT and the genetic basis of day-length adaptation underlying the postdomestication spread of maize. Proc Natl Acad Sci. 2012;109: E1913–E1921. doi:10.1073/pnas.1203189109

4. Abatzoglou JT. Development of gridded surface meteorological data for ecological applications and modelling. Int J Climatol. 2013;33: 121–131. doi:10.1002/joc.3413
