# Supplementary material for: Estimating parametric phenotypes that determine anthesis date in Zea mays: Challenges in combining ecophysiological models with genetics
Source: PLoS One. 2018 Apr 19;13(4):e0195841. doi: 10.1371/journal.pone.0195841 (PMC5909614; doi:10.1371/journal.pone.0195841)
Supplement: S2 File — (DOCX) [file pone.0195841.s002.docx]

A “Tie” is defined as the condition when two different parameter combinations result in identical predictions. For example, in Table A the two parameter sets shown have quite different values for P1, P2, and P2O. Nevertheless, the predicted anthesis dates for the two sets are exactly the same for all 11 site-years. For comparison, the rightmost column gives the observed anthesis dates obtained from the Panzea data repository (<http://www.panzea.org>) for line (Pop=10, LineNum=160). Clearly the same (and, for the line shown, optimal) RMSE value is obtained for both parameter sets, namely, 1.941 days.

Table A. Tied anthesis date prediction from two different parameter sets.

| Sites | Predicted anthesis dates Parameter set 1 | | | | Predicted anthesis dates Parameter set 2 | | | | Observed Pop=10, LineNum=160 |
| --- | --- | --- | --- | --- | --- | --- | --- | --- | --- |
|  | P1 | P2 | P2O | PHINT | P1 | P2 | P2O | PHINT |  |
|  | 255.51 | 1.101 | 10.98 | 28.493 | 286.19 | 1.599 | 13.84 | 28.439 |  |
| NY6 | 90 | | | | 90 | | | | 91.455 |
| NY7 | 83 | | | | 83 | | | | 82.0598 |
| MO6 | 70 | | | | 70 | | | | 69.3466 |
| MO7 | 70 | | | | 70 | | | | 69.8929 |
| NC6 | 67 | | | | 67 | | | | 68.4495 |
| NC7 | 71 | | | | 71 | | | | 66.4101 |
| IL6 | 75 | | | | 75 | | | | 77.31 |
| IL7 | 72 | | | | 72 | | | | 70.9998 |
| FL6 | 61 | | | | 61 | | | | 59.6886 |
| FL7 | 61 | | | | 61 | | | | 63.2252 |
| PR6 | 52 | | | | 52 | | | | 53.3623 |
