# Supplementary material for: Estimating parametric phenotypes that determine anthesis date in Zea mays: Challenges in combining ecophysiological models with genetics
Source: PLoS One. 2018 Apr 19;13(4):e0195841. doi: 10.1371/journal.pone.0195841 (PMC5909614; doi:10.1371/journal.pone.0195841)
Supplement: S3 File — (DOCX) [file pone.0195841.s003.docx]

**
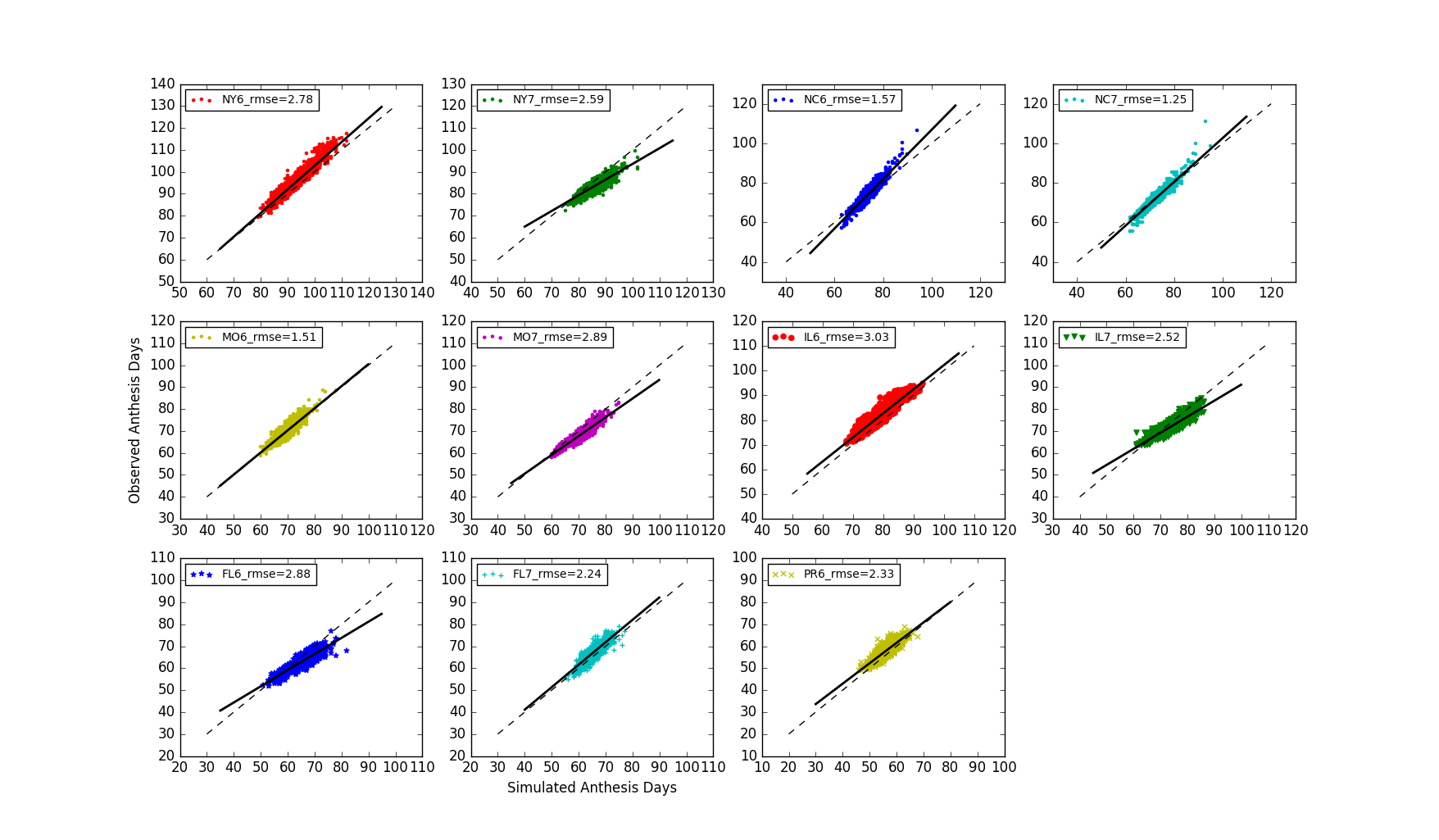
**

Fig. A. Simulated and observed anthesis date comparison for each site-years. Symbol color and marker is aligned with Fig 2 of manuscript.
