# Supplementary material for: Estimating parametric phenotypes that determine anthesis date in Zea mays: Challenges in combining ecophysiological models with genetics
Source: PLoS One. 2018 Apr 19;13(4):e0195841. doi: 10.1371/journal.pone.0195841 (PMC5909614; doi:10.1371/journal.pone.0195841)
Supplement: S4 File — (DOCX) [file pone.0195841.s004.docx]

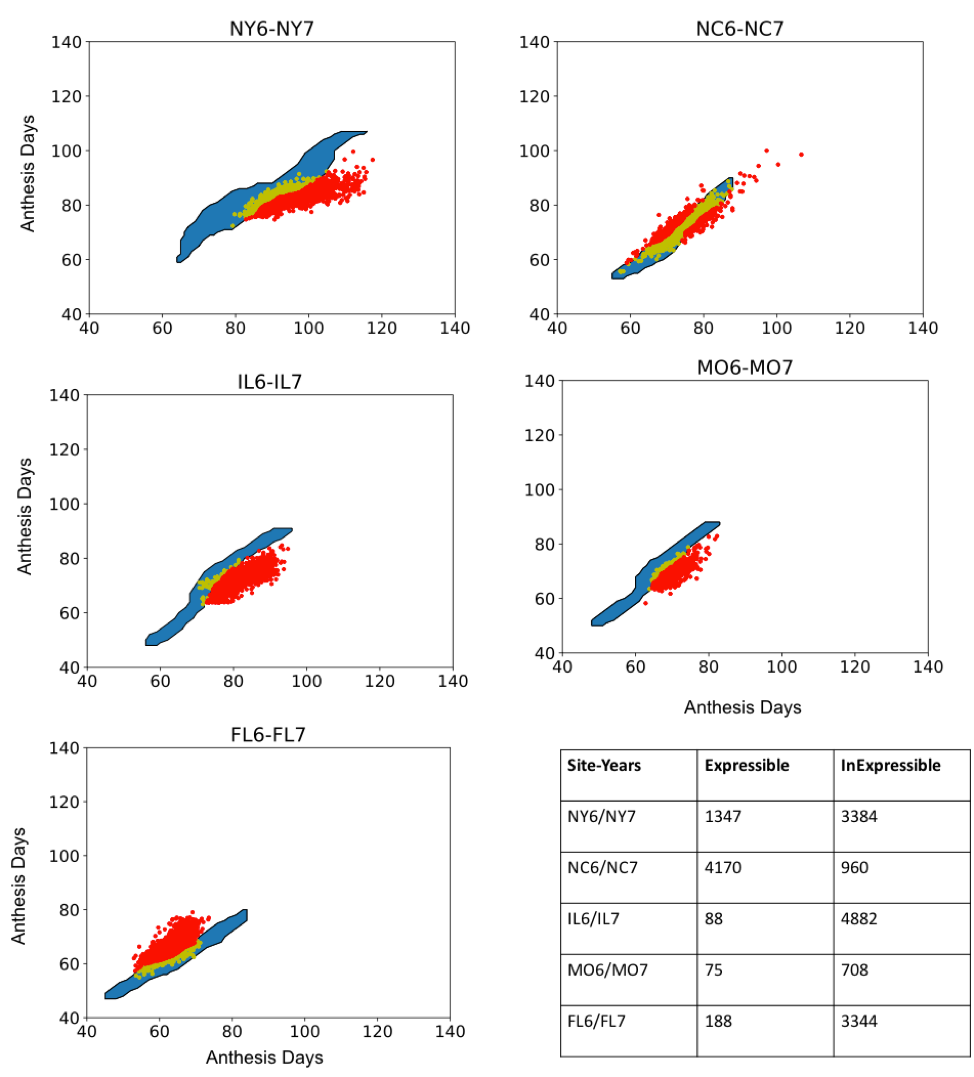


Fig A. Phenotype space plots for simulated and observed anthesis dates for five site-year pairs.  Blue regions outline simulated anthesis date pairs using six parameter scan Sobol database estimates. The data symbols depict expressible (yellow) and inexpressible (red) observed anthesis dates.

Table A. Ranges used for estimation of six parameters.

| **Parameter** | **Definition** | **Unit** | **Max** | **Min** | **No. of unique values** |
| --- | --- | --- | --- | --- | --- |
| P1 | Thermal time from seedling emergence to end of juvenile phase | GDD (^o^C) | 150 | 320 | 15001 |
| P2O | Critical Short day length below which daylength does not affect development rate | h | 11 | 13.5 | 4001 |
| P2 | Extent to which development (expressed as days) is delayed for each hour increase in photoperiod above the longest photoperiod at which development proceeds at a maximum rate (i.e, P2O) | rate | 0 | 1.2 | 2001 |
| PHINT | Phyllochron interval (Interval between successive leaf tip appearances) | GDD (^o^C) | 38 | 60 | 4501 |
| Tbase | Base temperature (Tbase) for development | (^o^C) | 6 | 12 | 4001 |
| Topt | Optimal temperature for development | (^o^C) | 26 | 36 | 6001 |


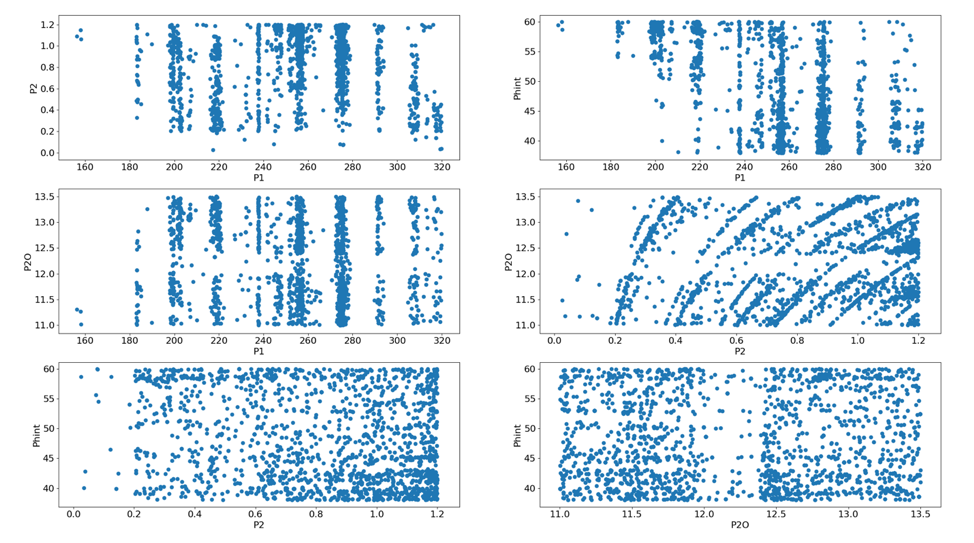


Fig B. Pairwise scatter plot for the parameter estimate resulting from six parameter runs.
